# A time series transcriptome analysis of cassava (*Manihot esculenta* Crantz) varieties challenged with Ugandan cassava brown streak virus

**DOI:** 10.1038/s41598-017-09617-z

**Published:** 2017-08-29

**Authors:** T. Amuge, D. K. Berger, M. S. Katari, A. A. Myburg, S. L. Goldman, M. E. Ferguson

**Affiliations:** 1 National Crops Resources Research Institute (NaCRRI), Namulonge, Uganda; 2 Department of Plant and Soil Sciences, Forestry and Agricultural Biotechnology Institute (FABI), University of Pretoria, Pretoria, South Africa; 3 International Institute of Tropical Agriculture (IITA), Nairobi, Kenya; 4 Center for Genomics and Systems Biology, New York University, New York, USA; 5 Genetics Department, Forestry and Agricultural Biotechnology Institute (FABI), University of Pretoria, Pretoria, South Africa

## Abstract

A time-course transcriptome analysis of two cassava varieties that are either resistant or susceptible to cassava brown streak disease (CBSD) was conducted using RNASeq, after graft inoculation with Ugandan cassava brown streak virus (UCBSV). From approximately 1.92 billion short reads, the largest number of differentially expressed genes (DEGs) was obtained in the resistant (Namikonga) variety at 2 days after grafting (dag) (3887 DEGs) and 5 dag (4911 DEGs). At the same time points, several defense response genes (encoding LRR-containing, NBARC-containing, pathogenesis-related, late embryogenesis abundant, selected transcription factors, chaperones, and heat shock proteins) were highly expressed in Namikonga. Also, defense-related GO terms of ‘translational elongation’, ‘translation factor activity’, ‘ribosomal subunit’ and ‘phosphorelay signal transduction’, were overrepresented in Namikonga at these time points. More reads corresponding to UCBSV sequences were recovered from the susceptible variety (Albert) (733 and 1660 read counts per million (cpm)) at 45 dag and 54 dag compared to Namikonga (10 and 117 cpm respectively). These findings suggest that Namikonga’s resistance involves restriction of multiplication of UCBSV within the host. These findings can be used with other sources of evidence to identify candidate genes and biomarkers that would contribute substantially to knowledge-based resistance breeding.

## Introduction

Cassava is among the six major crops of Africa, representing a staple food for >250 million people (FAO, 2010). Currently, production in parts of southern (Mozambique, Malawi and Angola), eastern (Uganda, Kenya, Tanzania, Rwanda and Burundi) and central Africa (D.R. Congo) is seriously affected by cassava brown streak disease (CBSD)^1, 2^. At least two virus species cause CBSD: *Cassava brown streak virus* (CBSV) and *Ugandan cassava brown streak virus* (UCBSV)^3, 4^. Here CBSVs is used to refer to both of these viruses. First reported in Tanzania, CBSD previously occurred at low levels primarily in coastal East Africa, Mozambique and around Lake Malawi and was thought to be restricted by altitude^5^. However in the early 2000s, CBSD had begun to spread around Lake Victoria, and by 2004, typical CBSD symptoms were widespread in farmers’ fields in central Uganda. The disease has spread steadily since then as far as DR Congo and South Sudan and now, together with cassava mosaic disease, causes over US$1 billion losses in production annually in Africa^1, 6, 7^.

Both CBSV and UCBSV are members of genus *Ipomovirus*, family *Potyviridae* ^3, 4, 8^. Both genomes have particles measuring 650 nm with pinwheel inclusions in their cells, distinctive of *Potyviridae* ^3^. The CBSV and UCBSV genomes are 9008 nucleotides (nt) and 9070 nt long, respectively^4, 8^. Each genome contains a single open reading frame that is translated into 2912 amino acids (aa) for CBSV and 2012 aa for UCBSV. Like other members of *Potyviridae*, both genomes encode the proteins P1, P3, 6K1, CI, 6K, VPg, Nla-Pro, Nlb and CP, with a new Ham1-like (Ham1) protein^3, 4, 8^. Neither genome encodes the helper-component protein (HC-Pro) that is encoded by other members of the family *Potyviridae* ^3, 4, 8^.

(U)CBSV is transmitted naturally by *Bemisia tabaci* ^5, 9, 10^ whiteflies in a semi-persistent manner, with the spiraling whitefly (*Aleurodicus dispersus*) being a possible alternative vector^11, 12^. The main form of disease spread in cassava fields in Africa is thought to be through virus-positive stem cuttings in this clonally propagated crop^13^. Mechanical transmission^14, 15^ and graft inoculation^9, 16^ are used in research studies.

**Figure 1:**
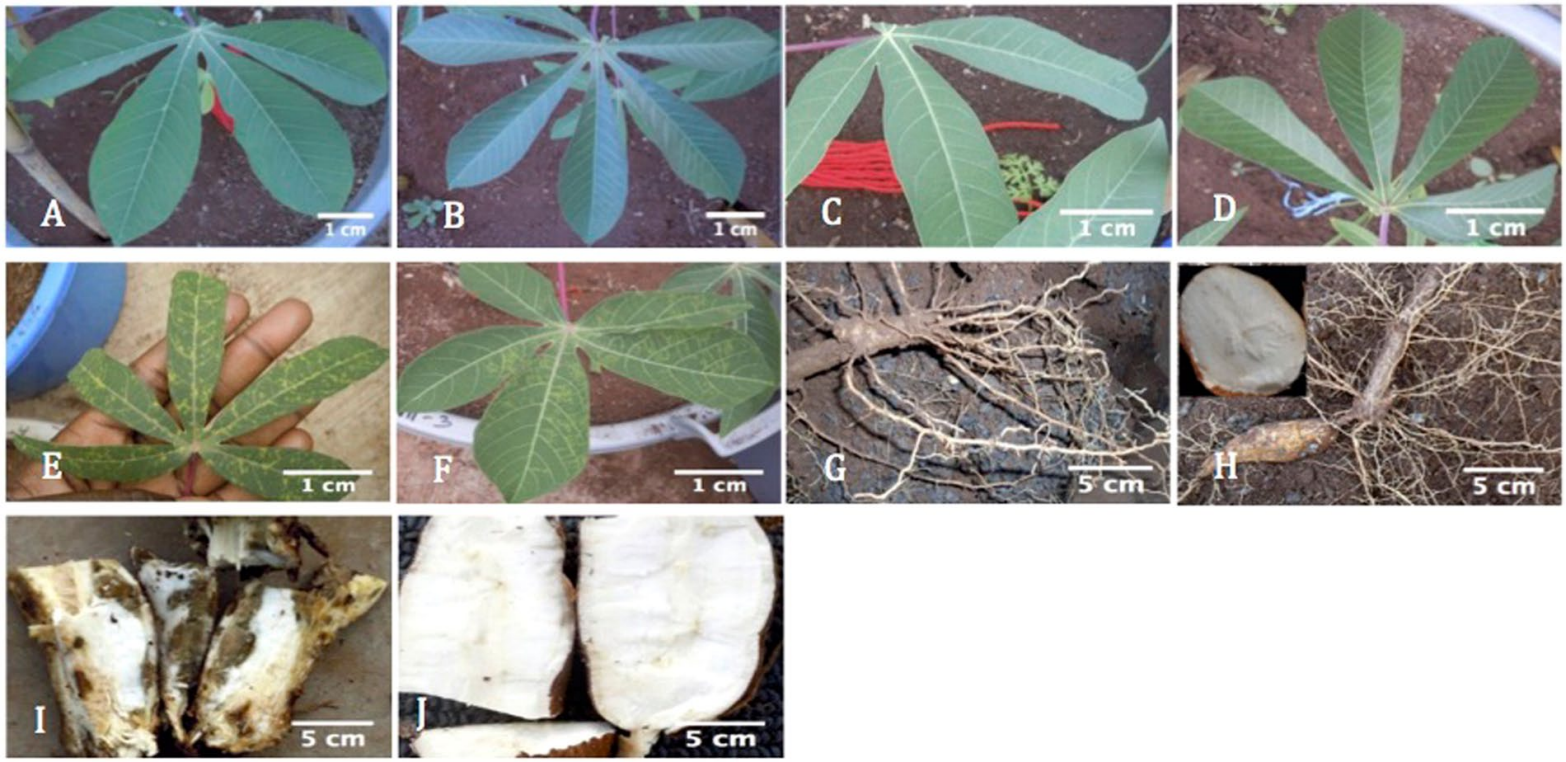
Leaf and root symptoms of UCBSV-inoculated versus mock-inoculated Albert and Namikonga plants during the experiment. (**A**) Albert, UCBSV inoculated, 8 dag; (**B**) Albert, mock inoculated, 8 dag; (**C**) Namikonga, UCBSV inoculated, 8 dag; (**D**) Namikonga, mock inoculated, 8 dag; (**E**) Albert, UCBSV inoculated, 54 dag; (**F**) Namikonga, UCBSV inoculated, 54 dag; (**G**) Albert root, UCBSV inoculated, 3 mag; (**H**) Namikonga root, UCBSV inoculated, 3 mag; (**I**) Albert root, UCBSV inoculated, 12 mag; (**J**) Namikonga root, UCBSV inoculated, 12 mag.

Infected susceptible plants develop chlorosis along leaf veins, brown streaks on stems, and root necrosis (Fig. 1), with severe infection causing shoot dieback. Dual infection with both virus species is common in farmers’ fields, although there are no reports of synergistic virus interaction^3, 4, 8, 17^, and both viruses cause similar symptoms, although those of CBSV tend to be more severe than UCBSV^18, 19^.

Currently, no known cassava variety has been reported to be immune to CBSVs, but varied levels of resistance or tolerance have been identified. Here, resistance is defined as the ability of the cassava variety to maintain a low virus load and show minimal shoot symptoms coupled with little or no root necrosis at harvest. Using this criteria, Namikonga (also known as Kaleso) was identified as resistant to CBSV from greenhouse experiments^20^ and classified as tolerant to CBSVs based on field symptoms and virus load in Uganda^21^. Cassava breeding is a lengthy process, and disease response is influenced by genotype-by-environment interactions^22^. To shorten the breeding cycle and improve the accuracy of variety selection, breeders are implementing genomics-based approaches. An understanding of the resistance mechanisms involved, including biochemical pathways, and the identification of candidate genes and biomarkers would contribute substantially to knowledge-based genomics breeding, including marker-assisted selection (MAS).

The use of RNAseq (RNA sequencing) has enabled the high-throughput identification of new genes, exons and exon junctions, splice variants and promoter regions in sequenced transcriptomes^23–25^. Previously, in cassava, an RNAseq-based transcriptome analysis of CBSD-resistant and -susceptible cassava varieties infected with CBSV was conducted to identify genes putatively involved in disease resistance; however, the results were inconclusive^20^.

In potyviruses, recessive resistance is known to involve >200 defense genes^26–28^. The majority of the cloned genes involve eukaryotic initiation factor (eIF) deployment^28–33^; however, other mechanisms involving defense response genes have been reported^34^. The eIF-mediated mechanism of resistance, also called ‘passive’^28^ or ‘loss-of-susceptibility’^35^ resistance, occurs when the host plant has a modified ribosomal protein sub-structure (usually eIF4E or eIF(iso)4E) so that the viral genome-linked (VPg) protein cap is unable to bind to the ribosome, thus preventing viral replication. An alternative mechanism of resistance to potyviruses was first identified in Arabidopsis plants infected with *Tobacco etch virus* (TEV) when a set of genes restricting the long-distance movement of TEV were cloned. These genes were named restricted TEV movement (RTM) genes. The RTM genes have since been found to cause resistance against other potyviruses including *Lettuce mosaic virus* (LMV) and *Plum pox virus* (PPV) by restricting the long-distance movement of virus particles^36^. Dominant resistance against potyviruses has been observed in pepper against *Potato virus Y* (PVY)^37^ and in *Solanum lycopersicum* against TEV and *Pepper mottle virus* (PMV)^29^. This dominant resistance may be characterized by a hypersensitive response (HR), as in tobacco resistant to PMV^38^ and *Tobacco mosaic virus* (TMV)^39^ and in potato resistant to both PVY and TEV^40^. The identification of potyvirus defense genes, such as those described above, motivated this study, as both CBSVs are potyviruses affecting cassava, a major food crop in sub-Saharan Africa.

The greater goal of this study was to define biomarkers for genomics-based breeding of CBSD-resistant cassava varieties to help address food insecurity in Africa. To improve our understanding of the mechanism of resistance and potentially identify candidate genes involved in resistance to UCBSV, DEGs were determined from a time-course experiment involving UCBSV-inoculated and mock-inoculated plants of two cassava varieties with contrasting responses to UCBSV infection, Albert and Namikonga. In addition, we tested the hypothesis that UCBSV accumulates at significantly lower rates in Namikonga compared to Albert because the induction of defense genes in Namikonga restricts virus replication^20, 21^.

## Results

### Symptoms of CBSD in leaves and roots

Characteristic CBSD symptoms were observed in UCBSV-inoculated plants and varied in magnitude by variety and time of observation. In the early sampling phase, no visible symptoms were observed on the leaves of either UCBSV-inoculated or mock-inoculated plants of both varieties (Fig. 1). In Albert, at the late sampling phase (54 dag), young leaves of UCBSV-inoculated plants showed chlorotic patterns along veins, expanding to form very large yellow areas (Fig. 1). At 12 months after grafting (mag), >50% of storage roots from UCBSV-inoculated Albert plants were necrotic, with a severity score of 4 (Fig. 1). Control Albert plants showed no disease symptoms on leaves and roots at 12 mag.

For Namikonga, leaf chlorotic spots (Fig. 1) were observed in the late sampling phase. At 54 dag, chlorosis covering 2–3 leaves per plant (score 2) was observed. At 12 mag, all storage roots were non-necrotic. The storage roots from mock-inoculated plants were also non-necrotic at 12 mag.

### Detection of UCBSV in UCBSV-inoculated cassava using RT-PCR and RNAseq

In the early phase, all samples (UCBSV- and mock-inoculated) were negative (non-detectable) for UCBSV except at 6 dag, when one of the ten UCBSV-inoculated plants of both the Albert and Namikonga varieties were positive for UCBSV using the RT-PCR assay (data not shown). Seven out of ten UCBSV-inoculated Albert plants were consistently positive at the late time phase (data not shown). For the Namikonga variety, seven out of ten UCBSV-inoculated plants tested positive for at least one sampling point during the late time phase (data not shown). However, this was not as consistent as the results from the Albert variety.

**Figure 2:**
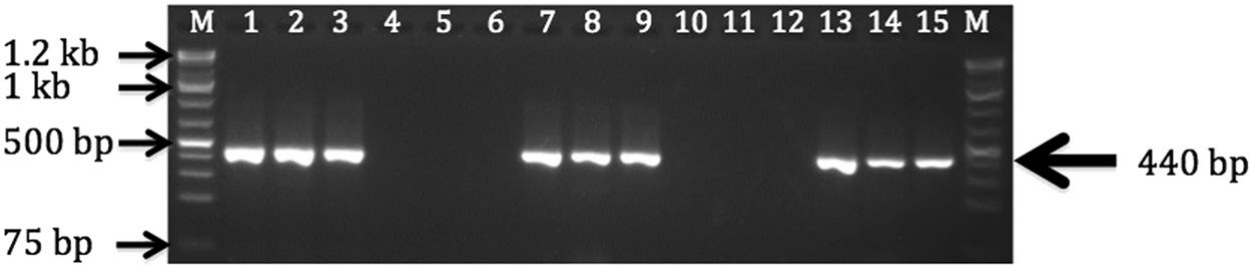
Diagnostic RT-PCR for UCBSV in the inoculum source NDL06/132, Albert and Namikonga plants at 3 mag. RNA from cassava plants from the UCBSV-inoculation experiment were amplified using the UCBSV diagnostic primers CBSDDF2/CBSDDR^54^, and the RT-PCR products were separated by agarose gel electrophoresis (2%). Lanes 1–3: UCBSV inoculum source (variety NDL06/132); lanes 4–6: Albert, mock grafted with virus-negative scions of NLD06/132; lanes 7–9: Albert, UCBSV inoculated; lanes 10–12: Namikonga, mock grafted with virus-negative scions of NLD06/132; lanes 13–15: Namikonga, UCBSV inoculated and M: 1 Kb+ ladder.

To confirm the presence of UCBSV in UCBSV-inoculated plants, leaves were sampled at 3 mag, after sampling for RNAseq at early and late time phases. End-point RT-PCR with the UCBSV-specific primers CBSDDF2/CBSDDR was performed on these 3 mag samples of Albert and Namikonga and 7 of the 10 plants of each variety tested were positive for UCBSV, having a 440-bp RT-PCR fragment when resolved on a 2% agarose gel (Fig. 2). Mock-inoculated plants had no visible RT-PCR fragment on the same gel, confirming the absence of UCBSV. An RT-PCR fragment putatively diagnostic for UCBSV (approximately 440 nt) was gel purified and Sanger sequenced before being aligned to the full UCBSV genome sequences from GenBank. The best alignment (98% identity, 100% query cover, E value = 3e179) was obtained with accession KF878103.1 (annotated as UCBSV). The same RT-PCR fragment size was amplified from the UCBSV inoculum source (variety NDL06/132), which indicates that the same virus species was present in the inoculum and UCBSV-inoculated plants (Fig. 2). Ten plants of each variety and each treatment were confirmed to be negative for UCBSV by RT-PCR prior to grafting. At 3 mag, after samples for RNAseq had been taken and frozen from all 40 plants at all time points, RT-PCR was conducted to determine whether they were UCBSV positive or not. Sanger sequencing of the RT-PCR products was performed to confirm the identity of UCBSV. Three of the UCBS-positive plants of each variety were randomly selected along with three mock-inoculated plants of each variety and used as biological replicates for the RNAseq study.

**Figure 3:**
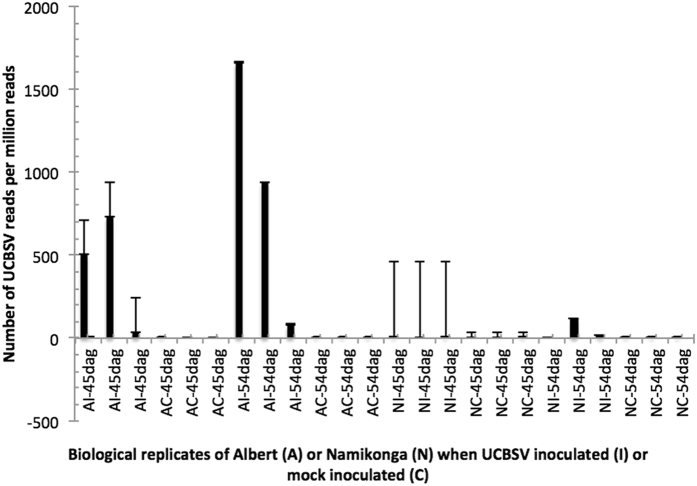
Number of UCBSV reads per million RNAseq reads retrieved from unmapped RNAseq reads of UCBSV inoculated (**I**) and control (**C**) samples from Albert (**A**) and Namikonga (**N**) varieties. In both varieties, UCBSV reads were only detected at 45 dag and 54 dag.

The number of UCBSV reads detected in each RNAseq sample at each time point is given in Supplementary Data S1. UCBSV sequences were detected in the inoculated susceptible variety Albert at 45 dag and 54 dag (late sampling phase). At 45 dag, 733, 507 and 37 UCBSV read counts per million (cpm) were detected from each of the three UCBSV-inoculated biological replicates of Albert (Fig. 3). At 54 dag, the same samples had 1660, 940 and 80 UCBSV read cpm. Surprisingly, one of the three mock-inoculated biological replicates of Albert had six UCBSV reads at 45 dag, and at 54 dag, two replicates had two UCBSV reads each, and one replicate had a single read. The surprising occurrence of UCBSV reads in mock-inoculated plants could be explained by contamination or the fact that the virus-negative plants host minute levels of UCBSV, which are not detectable by routine RT-PCR but are detectable with deep sequencing, such as RNAseq.

In Namikonga, very few UCBSV reads were detected in UCBSV-inoculated samples. The highest count (117 UCBSV sequences) was recorded in one biological replicate at 54 dag (Supplementary Data S1). This is 14 times lower than the highest count recorded in Albert at the same time point. The rest of the UCBSV-inoculated Namikonga samples had ≤10 UCBSV reads detected at any time point. For Namikonga, at 45 dag, one replicate from a mock-inoculated plant had one UCBSV sequence; the other two samples from mock-inoculated Namikonga plants had no detectable UCBSV sequence. The same was true for Namikonga at 54 dag.

### Read depth and mapping to the cassava reference genome (v4.1)

A total of approximately 1.92 billion raw reads were sequenced from 96 cDNA Namikonga and Albert libraries at eight time points with a coverage of approximately 20 million reads per library (Supplementary Table S1; Supplementary Data S2). All sequences have been deposited in NCBI’s Sequence Read Archive (SRA) SRP with BioProject ID PRJNA360340.

After sequence quality control (QC), including filtering based on Phred score and trimming, reads from each library were mapped to the cassava reference genome using Tophat2^41^. The cassava reference genome (version 4.1) is derived from a partial inbred line, AM560-2, a third generation self of the Latin American variety MCOL-1505^42^. Albert had 29,755,234,095 mapped reads and 8,231,859,633 unmapped reads, while Namikonga had 32,490,332,800 mapped and 8,477,326,725 unmapped reads (Supplementary Data S3). Therefore, Albert had 78% of its reads mapped and 22% unmapped to the cassava reference genome, while Namikonga had 79% mapped and 21% unmapped reads (Table 1). The cassava reference genome is derived from a partial inbred line, AM560-2, a third generation self of the Latin American variety MCOL-1505^42^. Supplementary Table S2 contains a summary of the per variety statistics after QC. Altogether, reads from 40 out of the 48 libraries sequenced at Dow AgroSciences (101 bp) (Supplementary Table S2b) mapped at least 84% to the cassava reference genome. Four samples mapped at 62.3–78.7%, while the remaining four mapped at 24–36.3%. Among the 50-bp libraries sequenced at UC Berkeley (Supplementary Table S2a), one sample mapped poorly (3.9%), another was 59.4% mapped, ten samples mapped between 74.1–79.7% and the remaining 36 libraries were >80% mapped to the reference genome. Out of the 96 sequenced cDNA libraries, all libraries that mapped poorly (<40%) also failed to cluster with respective biological replicates (see section ‘*Clustering of samples using data from RNAseq reads mapped to cassava genome*’). These outlier libraries were removed from subsequent analyses.

**Table 1:** Total statistics for RNAseq reads from cassava varieties Albert and Namikonga sampled at eight time points after mock or graft inoculation with UCBSV.

Time point	Mapping to cassava genome	Albert UCBSV inoculated	Albert Mock inoculated	Namikonga UCBSV inoculated	Namikonga Mock inoculated
Time zero	Mapped	—	4,218,021,147	—	3,996,224,040
	Unmapped	—	835,908,591	—	929,195,862
6 hag	Mapped	1,974,374,444	2,311,923,325	1,739,750,996	2,034,621,580
	Unmapped	465,562,984	558,691,687	373,228,177	416,264,064
1 dag	Mapped	1,782,476,393	1,606,541,112	1,751,611,492	1,415,764,268
	Unmapped	469,842,785	281,839,135	380,522,348	892,832,192
2 dag	Mapped	2,311,174,776	2,258,285,184	2,278,828,029	2,195,695,847
	Unmapped	425,576,288	411,150,949	494,433,874	648,333,549
5 dag	Mapped	1,152,362,044	900,243,552	2,223,072,359	1,809,373,734
	Unmapped	900,243,552	988,890,194	402,279,032	713,771,063
8 dag	Mapped	1,845,319,525	1,948,946,548	2,265,995,556	1,756,458,519
	Unmapped	352,441,534	373,483,677	493,741,019	547,890,775
45 dag	Mapped	1,701,360,044	1,258,543,240	2,269,455,275	2,155,716,341
	Unmapped	365,911,422	831,147,304	505,056,145	476,811,865
54 dag	Mapped	2,089,595,204	2,396,067,557	2,288,557,815	2,309,206,949
	Unmapped	473,446,221	497,723,310	545,859,978	657,106,782
**Total**	**Mapped**	**12**,**856**,**662**,**430**	**16**,**898**,**571**,**665**	**14**,**817**,**271**,**522**	**17**,**673**,**061**,**278**
	**Unmapped**	**3**,**453**,**024**,**786**	**4**,**778**,**834**,**847**	**3**,**195**,**120**,**573**	**5**,**282**,**206**,**152**

The time points were time zero (before graft inoculation), 6 hag, 1 dag, 2 dag, 5 dag, 8 dag, 45 dag and 54 dag. The figures reflect the number of RNAseq reads that mapped or did not map (unmapped) to the cassava reference genome v4.1^42^.

*HTseq-counts* ^43^ was used to count the number of reads that aligned to gene models of the cassava reference genome (v4.1). More than 22,000 out of 33,000 genes in the cassava reference genome v4.1 were represented by sequence information from either variety. Supplementary Data S4 summarizes the reads that could not be counted by HT-seq (i.e. reads that did not map to any gene models in the reference genome or reads that mapped to more than one gene model).

#### Clustering of samples using data from RNAseq reads mapped to the cassava genome

Using Pearson’s correlation, the normalized reads of samples from both Albert and Namikonga clustered by variety (Supplementary Figure S1). Respective biological replicates clustered together by time point, variety and treatment (UCBSV inoculated and mock inoculated), irrespective of the laboratory where samples had been sequenced. This was expected, as RNAseq samples sequenced from different laboratories are comparable provided that recommended laboratory procedures are followed and sequence reads are filtered appropriately^44^. However, ten of 96 samples were outliers, and these were removed from further analyses (Supplementary Table S3). Seven of these removed outlier samples were from Albert (1dag_Alb_Inf_2, 5dag_Alb_Inf_1, 1dag_Alb_Ctl_3, 2dag_Alb_Ctl_3, 5dag_Alb_Ctl_2, 8dag_Alb_Ctl_1 and 45_dag_Alb_Ctl_1) (Supplementary Figure S1a), and three were from Namikonga (2dag_Nam_Ctl_2, 54dag_Nam_Ctl_3 and 6hag_Nam_Ctl_2) (Supplementary Figure S1b).

In Albert, once the outlier samples were removed and reads re-normalized, the median gene expression values were comparable across all time points (Supplementary Figure S2a). The treatments had a distinct median range of filtered, normalized reads cutting across UCBSV-inoculated and mock-inoculated treatments (approximately 5.8–6.2). The median of the gene expression values in the 1 dag and 45 dag samples was slightly lower (5.8–6.0) compared to other time points (6.0–6.2).

For Namikonga, the median of the re-normalized gene expression values was within the same range (approx. 6.5) (Supplementary Figure S2b), as was the number of outlier genes above the upper quartile range. A slightly higher number of outlier genes were recorded at 45 dag, and the reverse at 54 dag. Samples with medians below the lower quartile were only observed at 6 hag (one UCBSV-inoculated and one mock-inoculated sample), 8 dag (three UCBSV-inoculated and one mock-inoculated sample) and 54 dag (two UCBSV-inoculated and mock-inoculated samples). After checking the distribution of the data, DESeq^45^ was used to identify DEGs (Supplementary Data S5) using at least two biological replicates per treatment at each time point (Supplementary Table S3).

### Identification of DEGs between mock- and UCBSV-inoculated samples of susceptible (Albert) and resistant (Namikonga) varieties at different time points

**Figure 4:**
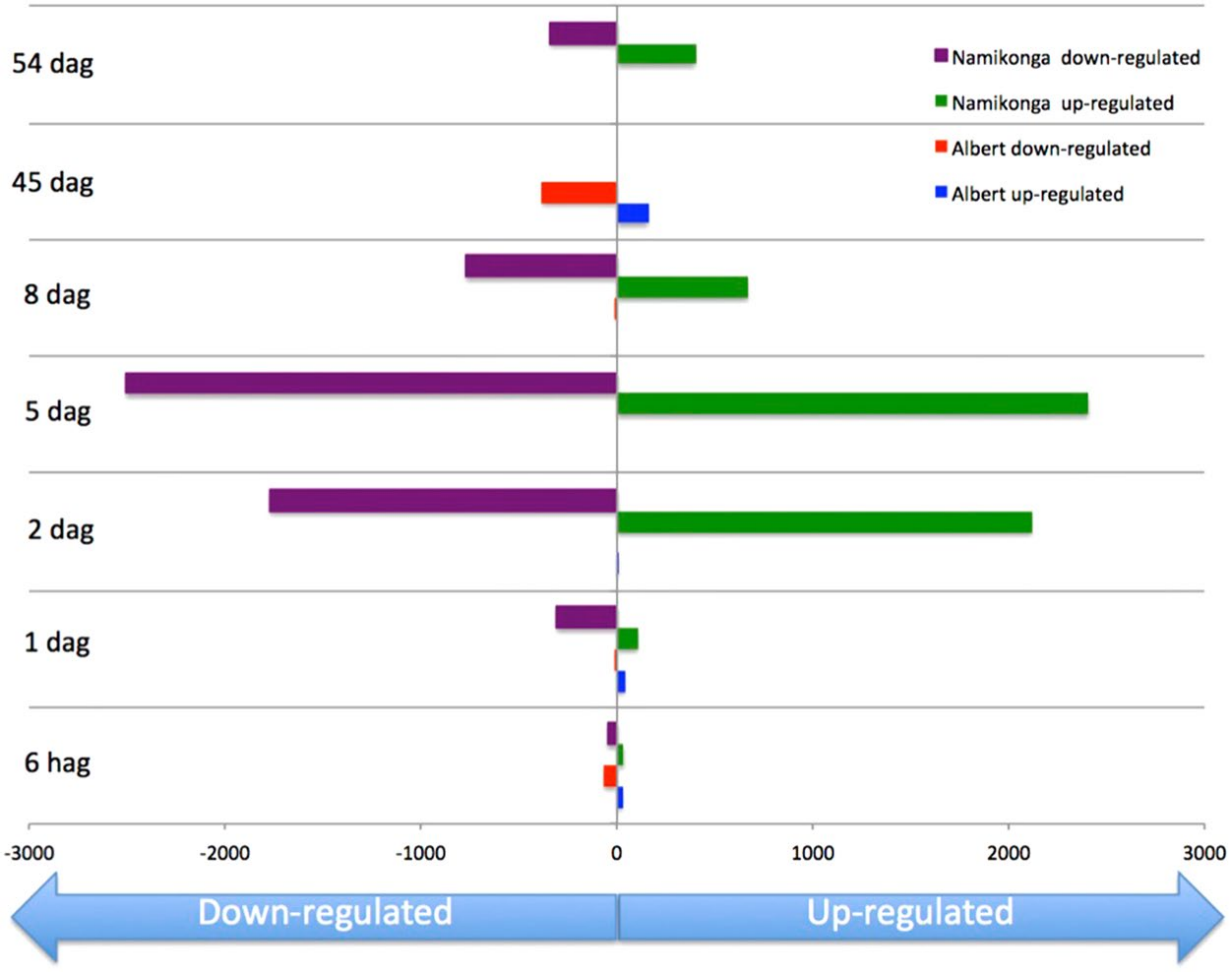
Number of DEGs between UCBSV-inoculated and mock-inoculated cassava varieties Albert and Namikonga at 6 hag, 1 dag, 2 dag, 5 dag, 8 dag, 45 dag and 54 dag. The DEGs were identified using the DESeq method^45^.

To identify DEGs, UCBSV-inoculated samples were compared with mock-inoculated samples at each time point, per variety. We defined DEGs as those that were computed by DESeq to have a false discovery rate (FDR) for differential expression of less than 10% for a particular treatment comparison. We did not apply an additional filter of log2 fold change (log2FC) values, but DEGs with positive or negative log2FC values were classified as up-regulated (log2FC ≥ 0), or down-regulated (log2FC ≤ 0) for a treatment, respectively. In total, more genes were differentially expressed at particular time points in Namikonga (10,028) compared to Albert (688) (Fig. 4). It is unlikely that this is due to sequence differences between these varieties and the reference genome, since both had a similar proportion of unmapped reads (approximately 21%) (Table 1). For Namikonga, there were approximately equal numbers of up- and down-regulated genes at all time points, except 1 dag, when only 26% of the DEGs were up-regulated. For Albert, more than 75% of the DEGs were up-regulated at 1 dag and 2 dag, whereas there were more down-regulated genes at the other time points.

In Namikonga, the greatest differential gene expression (either down-regulated or up-regulated) occurred at the early time points, particularly 2 dag (3887 DEGs), 5 dag (4911 DEGs) and 8 dag (1438 DEGs) (Fig. 4). The number of DEGs in Albert in the early time phase was much lower than in Namikonga, with the highest being at 6 hag with only 92 DEGs. At the late time points, few genes were differentially expressed in either variety. Albert had 543 DEGs at 45 dag (which was the maximum for Albert) and none at 54 dag. Namikonga had no DEGs at 45 dag but 738 at 54 dag (Fig. 4). All statistically significant DEGs were further characterized by functional annotation and GO term enrichment.

### GO term enrichment of DEGs

Over-represented GO terms were identified among the up-regulated and down-regulated genes separately at each time point and in each variety. The numbers of enriched GO terms for each treatment are presented in Supplementary Table S4. Namikonga had the largest number of DEGS at 2 dag (3887 DEGs) and 5 dag (4911 DEGs), which also corresponded to the largest number of over-represented GO terms (Supplementary Table S4). For this reason, these time points were the focus for further analysis, particularly the identification of enriched defense-related GO terms.

### Enriched GO terms in Namikonga that are related to defense responses

**Figure 5:**
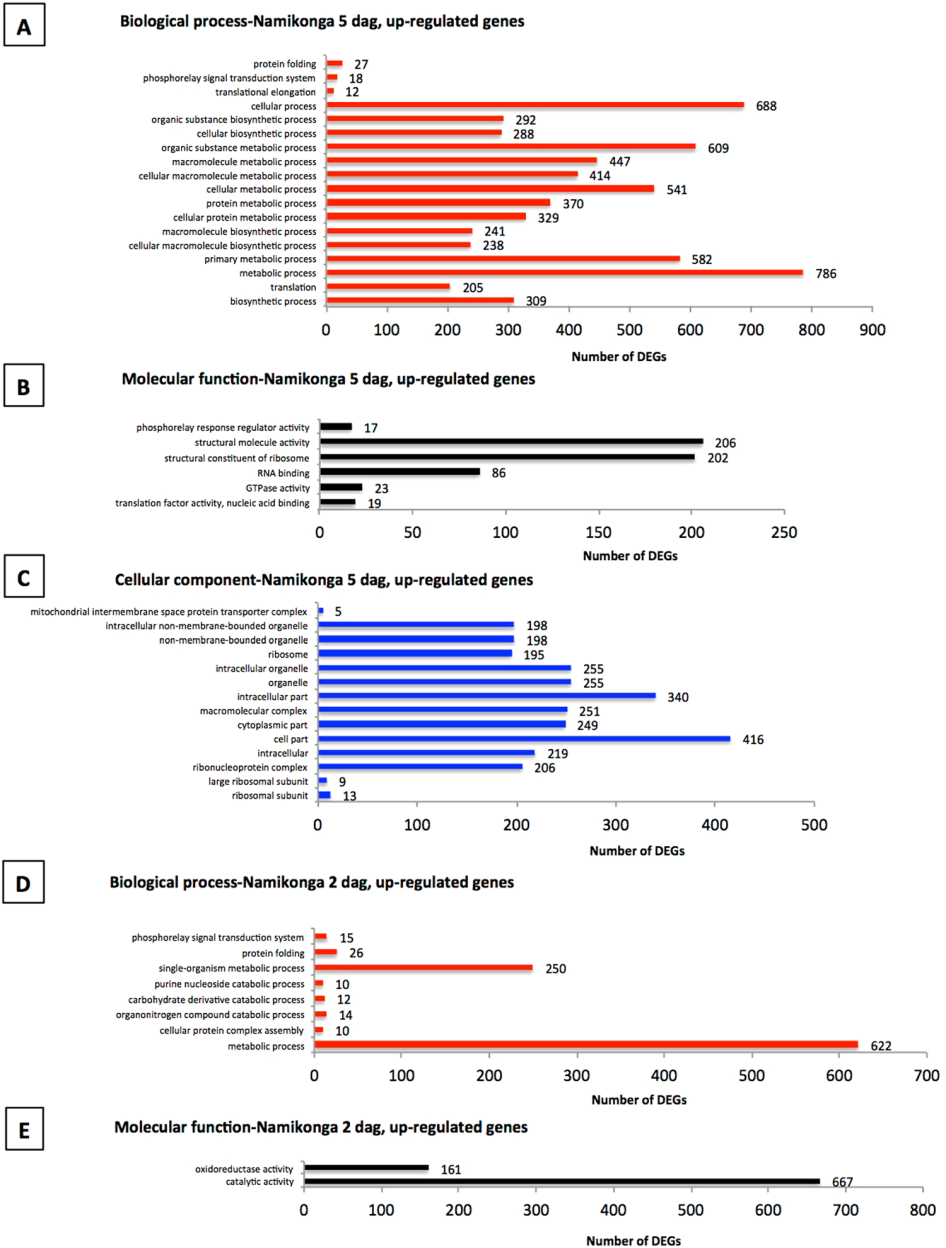
Over-represented GO terms for up-regulated genes in Namikonga at 2 dag and 5 dag. **A** = Over-represented GO terms of the category “biological process” in the Namikonga up-regulated genes at 5 dag; **B** = Over-represented GO terms of the category “molecular function” in the Namikonga up-regulated genes at 5 dag; **C** = Over-represented GO terms of the category “cellular component” in the Namikonga up-regulated genes at 5 dag; **D** = Over-represented GO terms of the category “biological process” in the Namikonga up-regulated genes at 2 dag; **E** = Over-represented GO terms of the category “molecular function” in the Namikonga up-regulated genes at 2 dag. Results are presented for over-represented GO terms with adjusted p-values in the 95% confidence interval.

Among the over-represented GO terms of Namikonga identified at 2 dag and 5 dag (Fig. 5), the following terms that are likely to be related to pathogen defense^33, 46, 47^ were identified: translational elongation (GO:0006414), translation factor activity, nucleic acid binding (GO:0008135), ribosomal subunit (GO:0044391) and phosphorelay signal transduction (GO:0000160 and GO:0000156) were each represented by 12, 19, 13 and 18 (17 in GO:0000156) DEGs, respectively (Table 2). None of these defense-related GO terms were over-represented in Albert. Individual genes with these GO terms were significantly differentially expressed in Namikonga, but not in Albert. The expression of genes with these defense-related GO terms was further examined.

**Table 2:** GO term over-representation analysis of the up-regulated genes of Namikonga at 2 dag and 5 dag.

GO ID	Enriched GO terms found within the up-regulated genes of Namikonga at 2 dag	No. of DEGs	Adj. p-value
	***Biological process***		
GO:0043623	Cellular protein complex assembly	10	2.00E-02
GO:0006152	Purine nucleoside catabolic process	10	5.E-03
GO:1901136	Carbohydrate derivative catabolic process	12	2.E-02
GO:1901565	Organonitrogen compound catabolic process	14	1.E-02
**GO:0000160**	**Phosphorelay signal transduction system**	**15**	**2**.**E-02**
GO:0006457	Protein folding	26	5.E-03
GO:0044710	Single-organism metabolic process	250	1.E-02
GO:0008152	Metabolic process	622	1.E-02
	***Molecular function***		
GO:0016491	Oxidoreductase activity	161	3.E-02
GO:0003824	Catalytic activity	667	9.E-04
	**Enriched GO terms found within the up-regulated genes of Namikonga at 5 dag**		
	***Biological process***		
**GO:0006414**	**Translational elongation**	**12**	**3**.**E-03**
**GO:0000160**	**Phosphorelay signal transduction system**	**18**	**1**.**E-02**
GO:0006457	Protein folding	27	3.E-02
GO:0006412	Translation	205	3.E-68
GO:0034645	Cellular macromolecule biosynthetic process	238	1.E-59
GO:0009059	Macromolecule biosynthetic process	241	5.E-56
GO:0044249	Cellular biosynthetic process	288	3.E-43
GO:1901576	Organic substance biosynthetic process	292	1.E-42
GO:0009058	Biosynthetic process	309	5.E-40
GO:0044267	Cellular protein metabolic process	329	2.E-15
GO:0019538	Protein metabolic process	370	1.E-10
GO:0044260	Cellular macromolecule metabolic process	414	4.E-15
GO:0043170	Macromolecule metabolic process	447	3.E-11
GO:0044237	Cellular metabolic process	541	4.E-18
GO:0044238	Primary metabolic process	582	2.E-10
GO:0071704	Organic substance metabolic process	609	3.E-11
GO:0009987	Cellular process	688	6.E-13
GO:0008152	Metabolic process	786	1.E-04
	***Molecular function***		
**GO:0000156**	**Phosphorelay response regulator activity**	**17**	**5**.**E-03**
**GO:0008135**	**Translation factor activity**, **nucleic acid binding**	**19**	**1**.**E-02**
GO:0003924	Gtpase activity	23	5.E-03
GO:0003723	RNA binding	86	4.E-12
GO:0003735	Structural constituent of ribosome	202	3.E-78
GO:0005198	Structural molecule activity	206	3.E-73
	***Cellular component***		
GO:0042719	Mitochondrial intermembrane space protein transporter complex	5	3.E-02
GO:0015934	Large ribosomal subunit	9	4.E-03
**GO:0044391**	**Ribosomal subunit**	**13**	**3**.**E-04**
GO:0005840	Ribosome	195	4.E-63
GO:0043228	Non-membrane-bounded organelle	198	9.E-56
GO:0030529	Ribonucleoprotein complex	206	3.E-64
GO:0005622	Intracellular	219	3.E-19
GO:0044444	Cytoplasmic part	249	1.E-47
GO:0032991	Macromolecular complex	251	9.E-28
GO:0043226	Organelle	255	2.E-21
GO:0044424	Intracellular part	340	2.E-14
GO:0044464	Cell part	416	9.E-09

#### Translation initiation in the ribosomal subunit

The role of eIF4E genes in Potyvirus resistance has been demonstrated in many plants^28^. We identified two eIF4E genes (Cassava4.1_016620 m.g and Cassava4.1_013223 m.g) for which reads were mapped in our dataset. Neither gene was significantly differentially expressed in our experiment, except for Cassava4.1_016620 m.g in Namikonga at a single time point (5 dag) (log2FC = 0.72; adj. p-value = 0.02) (Supplementary Table S5). The eIF4E gene Cassava4.1_013223 m.g^20^ had a log2FC of −0.17 and 0.63 at 2 dag and 5 dag in Namikonga. This gene was up-regulated by a log2FC of 0.1 and 0.4 in Albert at 2 dag and 5 dag, which was not statistically significant.

Although the data did not show transcriptional differences in eIF4E, there were three main enriched GO terms (GO:0006414, GO:0008135 and GO:0044391) associated with translation in Namikonga at the early time points. This indicates, in general, the potential role(s) of translation in CBSD resistance in Namikonga, and therefore individual genes are reported here. Within these GO terms, Namikonga had several families of translation factors, including eIF4E, EF1B, EF-Ts, 5A-1, eIF3 subunit 7, eIF2 subunit 1, 3B1, and IF2/1F5, each having one or two genes identified at 2 dag and/or 5 dag (Table 3). The two families of EF-T genes (5A-1 and GTP binding EF-Tu) had a log2FC ≤ 1 in both varieties, yet were statistically significant and up-regulated in Namikonga, in contrast to Albert. The EF1B gene was up-regulated in Namikonga, but the results were not significant in either variety. Within the same three GO terms, genes encoding ribosomal protein L10 and 60 S acidic ribosomal proteins were statistically significantly up-regulated in Namikonga, but non-significantly down-regulated in Albert (Table 3).

**Table 3:** Comparison of UCBSV-induced genes corresponding to the over-represented GO terms ‘translational elongation’, ‘translation factor activity, nucleic acid binding’, and ‘ribosomal subunit’ at 2 and 5 dag in Namikonga and Albert.

Gene ID	Gene annotation	2 dag		5 dag	
		Albert	Namikonga	Albert	Namikonga
		Log 2 FC (Adj.Pv)	Log 2 FC (Adj.Pv)	Log 2 FC (Adj.Pv)	Log 2 FC (Adj.Pv)
**GO term: Translational elongation (GO:0006414)**					
Cassava4.1_015319 m.g	Translation elongation factor EF1B/ribosomal protein S6 family protein	0.0(1)	−0.7(1.3E-01)	0.2(1)	0.6(6.8E-02)
Cassava4.1_010349 m.g	Translation elongation factor Ts (EF-Ts), putative	−0.4(1)	−0.5(5.7E-01)	0.5(1)	1.0(1.3E-02)
Cassava4.1_018059 m.g	Eukaryotic elongation factor 5A-1	0.0(1)	0.5(3.1E-01)	−0.9(1)	0.9(6.0E-04)
Cassava4.1_018052 m.g	Eukaryotic elongation factor 5A-1	−0.2(1)	0.2(8.1E-01)	−0.1(1)	0.9(5.5E-03)
Cassava4.1_007378 m.g	GTP binding Elongation factor Tu family protein	−0.2(1)	0.6(3.1E-01)	−0.3(1)	1.7(4.5E-05)
Cassava4.1_011934 m.g	Ribosomal protein L10 family protein	−0.1(1)	−0.2(8.7E-01)	−0.4(1)	0.8(7.5E-03)
Cassava4.1_019599 m.g	60 S acidic ribosomal protein family	0.0(1)	−0.5(4.4E-01)	−0.6(1)	1.9(6.7E-10)
Cassava4.1_019305 m.g	60 S acidic ribosomal protein family	0.0(1)	−0.1(1.0E + 00)	−0.7(1)	0.9(3.7E-03)
Cassava4.1_020132 m.g	60 S acidic ribosomal protein family	0.1(1)	−0.2(8.2E-01)	−0.9(1)	0.7(1.5E-02)
Cassava4.1_019568 m.g	60 S acidic ribosomal protein family	−0.1(1)	−0.2(8.9E-01)	0(1)	0.6(4.4E-02)
Cassava4.1_019588 m.g	60 S acidic ribosomal protein family	0.0(1)	−0.1(9.4E-01)	−0.5(1)	0.8(4.2E-03)
Cassava4.1_018344 m.g	60 S acidic ribosomal protein family	0.0(1)	0.2(7.2E-01)	−0.7(1)	1.0(5.3E-04)
**GO term: Translation factor activity**, **nucleic acid binding (GO:0008135)**					
Cassava4.1_016620 m.g	Eukaryotic initiation factor 4E protein	0.2(1)	−0.2(8.6E-01)	0.4(1)	0.7(2.0E-02)
Cassava4.1_013223 m.g	Eukaryotic translation initiation factor 4E	0.1(1)	−0.2(9.1E-01)	0.4(1)	0.6(6.2E-02)
Cassava4.1_004498 m.g	Eukaryotic translation initiation factor 3 subunit 7 (eIF3)	−0.1(1)	−0.4(5.0E-01)	0.1(1)	0.8(1.0E-02)
Cassava4.1_033528 m.g	Eukaryotic initiation factor 3 gamma subunit family protein	−0.1(1)	0.9(5.8E-01)	1.7(1)	1.6(9.2E-02)
Cassava4.1_011050 m.g	Eukaryotic translation initiation factor 2 subunit 1	0.0(1)	−0.5(3.2E-01)	−0.6(1)	0.7(2.4E-02)
Cassava4.1_017333 m.g	Eukaryotic initiation factor 3 gamma subunit family protein	−0.2(1)	0.7(3.1E-01)	0.8(1)	1.5(1.0E-03)
Cassava4.1_018052 m.g	Eukaryotic elongation factor 5A-1	−0.2(1)	0.2(8.1E-01)	−0.1(1)	0.9(5.5E-03)
Cassava4.1_018059 m.g	Eukaryotic elongation factor 5A-1	0.0(1)	0.5(3.1E-01)	−0.9(1)	0.9(6.0E-04)
Cassava4.1_001203 m.g	Eukaryotic translation initiation factor 3 C	−0.2(1)	−0.5(3.2E-01)	0.3(1)	1.5(2.0E-07)
Cassava4.1_020990 m.g	Eukaryotic release factor 1–3	−0.1(1)	−0.4(5.7E-01)	−0.2(1)	0.6(7.8E-02)
Cassava4.1_002530 m.g	Translation initiation factor 3B1	0.1(1)	−0.1(9.7E-01)	−0.3(1)	0.8(1.3E-02)
Cassava4.1_002467 m.g	Translation initiation factor 2, small GTP-binding protein	0.0(1)	−0.3(7.4E-01)	0.6(1)	0.6(1.1E-01)
Cassava4.1_015319 m.g	Translation elongation factor EF1B/ribosomal protein S6 family protein	0.0(1)	−0.7(1.3E-01)	0.2(1)	0.6(6.8E-02)
Cassava4.1_007596 m.g	Translation initiation factor IF2/IF5	−0.1(1)	0.1(9.9E-01)	−0.4(1)	0.7(1.5E-02)
Cassava4.1_007700 m.g	Translation initiation factor IF2/IF5	0.1(1)	−0.6(1.7E-01)	−0.1(1)	0.6(4.3E-02)
Cassava4.1_010349 m.g	Translation elongation factor Ts (EF-Ts), putative	−0.4(1)	−0.5(5.7E-01)	0.5(1)	1.0(1.3E-02)
Cassava4.1_007378 m.g	GTP binding Elongation factor Tu family protein	−0.2(1)	0.6(3.1E-01)	−0.3(1)	1.7(4.5E-05)
Cassava4.1_018593 m.g	Nucleic acid-binding, OB-fold-like protein	0.2(1)	−0.6(3.0E-01)	0(1)	0.9(5.1E-02)
Cassava4.1_005116 m.g	Peptide chain release factor 2	−0.1(1)	1.2(4.5E-01)	1.6(1)	2.0(8.2E-04)
**GO term: Ribosomal subunit (GO:0006414)**					
Cassava4.1_018516 m.g	Translation protein SH3-like family protein	−0.1(1)	−0.2(8.7E-01)	−0.5(1)	0.5(9.9E-02)
Cassava4.1_016258 m.g	Ribosomal protein 5B	−0.2(1)	−0.4(4.7E-01)	0.2(1)	1.0(2.2E-04)
Cassava4.1_013486 m.g	Ribosomal protein l18e/L15 superfamily protein	−0.1(1)	0.5(4.8E-01)	−0.2(1)	0.8(8.6E-02)
Cassava4.1_015972 m.g	Ribosomal protein l1p/L10e family	−0.2(1)	0.3(6.2E-01)	−0.4(1)	1.1(7.9E-05)
Cassava4.1_015937 m.g	Ribosomal protein l1p/L10e family	−0.1(1)	−0.1(9.8E-01)	0.1(1)	0.8(3.6E-03)
Cassava4.1_009639 m.g	Ribosomal protein l1p/L10e family	0.3(1)	0.4(7.4E-01)	0.4(1)	2.0(9.5E-08)
Cassava4.1_009636 m.g	Ribosomal protein l1p/L10e family	0.4(1)	0.0(1.0E + 00)	0.3(1)	1.5(9.8E-05)
Cassava4.1_008473 m.g	Ribosomal protein l1p/L10e family	−0.1(1)	−0.8(8.2E-02)	0.4(1)	0.7(3.6E-02)
Cassava4.1_017425 m.g	Ribosomal protein l22p/L17e family protein	−0.2(1)	−0.2(8.3E-01)	−0.5(1)	1.1(8.8E-05)
Cassava4.1_019192 m.g	Ribosomal protein l22p/L17e family protein	0.0(1)	−0.4(5.2E-01)	−0.4(1)	1.2(2.0E-05)
Cassava4.1_012305 m.g	40 s ribosomal protein SA	−0.3(1)	−0.1(1.0E + 00)	1.1(1)	1.8(7.2E-08)
Cassava4.1_012175 m.g	40 s ribosomal protein SA	−0.3(1)	−0.2(8.8E-01)	0.6(1)	1.0(2.4E-03)
Cassava4.1_012280 m.g	40 s ribosomal protein SA	−0.3(1)	0.0(1.0E + 00)	1.7(1)	1.3(6.4E-04)

#### Phosphorelay signal transduction and response regulation

There were two GO terms associated with phosphorelay signal transduction: phosphorelay signal transduction system (GO:0000160) and phosphorelay response regulator activity (GO:0000156). The first GO term contained 18 genes that encoded histidine-related proteins and response regulators. All 18 genes were up-regulated in Namikonga (log2FC of 0.8–3.5), but in Albert, 12 genes were down-regulated, while the other six were expressed at very low levels (log2FC of 0.0–0.8). The other GO term, phosphorelay response regulator activity, had 17 genes that were expressed in the same pattern as the first GO term (GO:0000160) (Table 4).

**Table 4:** Comparison of UCBSV-induced genes corresponding to the over-represented GO terms ‘phosphorelay signal transduction system’ and ‘response regulator activity’ at 2 and 5 dag in Namikonga and Albert.

Gene ID	Gene annotation	2 dag		5 dag	
		Albert	Namikonga	Albert	Namikonga
		Log 2 FC (Adj.Pv)	Log 2 FC (Adj.Pv)	Log 2 FC (Adj.Pv)	Log 2 FC (Adj.Pv)
**GO term: Phosphorelay signal transduction system (GO:0000160) and response regulator activity (GO:0000156)**					
Cassava4.1_000395 m.g	Histidine kinase 2	0.1(1)	1.3(6.2E-04)	−0.9(1)	2.1(8.6E-14)
Cassava4.1_000859 m.g	CHASE domain containing histidine kinase protein	0.1(1)	2.6(3.3E-06)	−0.3(1)	2.1(1.6E-11)
Cassava4.1_001670 m.g	Histidine kinase 2	0.8(1)	4.7(1.6E-36)	0.1(1)	2.3(3.7E-18)
Cassava4.1_002119 m.g	Pseudo-response regulator 7	0.3(1)	1.8(1.0E-07)	−0.1(1)	0.8(3.8E-03)
Cassava4.1_002915 m.g	Response regulator 12	0.6(1)	1.5(1.7E-05)	0.1(1)	2.4(1.1E-10)
Cassava4.1_015446 m.g	Response regulator 9	0.8(1)	4.6(1.3E-04)	−1.5(1)	3.5(1.4E-14)
Cassava4.1_015815 m.g	Response regulator 9	−0.2(1)	4.0(6.9E-24)	−1.5(1)	1.8(2.3E-01)
Cassava4.1_018229 m.g	Histidine-containing phosphotransmitter 1	0.4(1)	1.0(3.0E-01)	−0.5(1)	1.4(4.1E-07)
Cassava4.1_018306 m.g	Histidine-containing phosphotransmitter 1	−0.4(1)	3.2(7.0E-07)	0.8(1)	−1.1(5.0E-01)
Cassava4.1_022288 m.g	Response regulator 9	0.9(1)	4.1(1.6E-01)	0.3(1)	2.5(8.7E-08)
Cassava4.1_022850 m.g	Pseudo-response regulator 7	0.2(1)	4.4(1.0E-14)	−0.5(1)	2.5(4.5E-08)
Cassava4.1_024410 m.g	Histidine kinase 5	−0.2(1)	3.8(1.9E-02)	2.6(1)	0.1(1.0E + 00)
Cassava4.1_025220 m.g	Pseudo-response regulator 3	0.7(1)	4.3(2.5E-16)	−0.5(1)	1.2(1.7E-03)
Cassava4.1_026975 m.g	Response regulator 3	0.4(1)	4.1(3.0E-03)	0.0(1)	3.5(1.3E-12)
Cassava4.1_033332 m.g	Pseudo-response regulator 9	0.4(1)	1.3(5.6E-04)	−0.7(1)	1.0(2.9E-04)
Cassava4.1_002375 m.g	Signal transduction histidine kinase, hybrid-type, ethylene sensor	−0.6(1)	−2.6(4.8E-06)	−0.2(1)	0.8(1.2E-02)
Cassava4.1_015797 m.g	Response regulator 5	−0.4(1)	1.5(5.0E-01)	−1.3(1)	1.3(1.6E-02)
Cassava4.1_023472 m.g	Response regulator 3	−0.7(1)	0.2(9.4E-01)	0.6(1)	1.4(3.8E-03)
Cassava4.1_027609 m.g	Response regulator 9	0.7(1)	0.2(9.6E-01)	−0.4(1)	1.8(2.3E-07)
Cassava4.1_027924 m.g	Signal transduction histidine kinase, hybrid-type, ethylene sensor	0.1(1)	0.2(8.3E-01)	0.8(1)	0.8(5.2E-02)
Cassava4.1_028820 m.g	Response regulator 2	0.2(1)	1.4(8.4E-02)	−0.2(1)	1.4(4.8E-02)

### Specific defense response genes are highly expressed in Namikonga at specific time points

**Figure 6:**
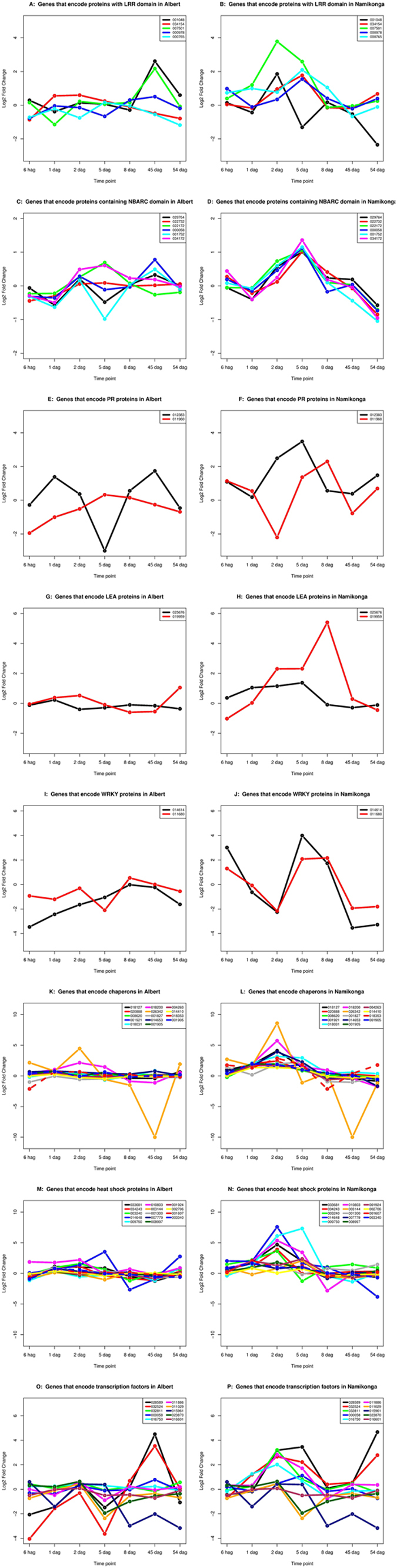
Selected DEGs that were expressed at significantly different levels in susceptible (Albert) and resistant (Namikonga) varieties at different time points (RNAseq data). Gene families include **A** and **B** = Leucine-rich repeat (LRR)-containing proteins, **C** and **D** = NBARC-containing proteins, **E** and **F** = Pathogenesis-related (PR) proteins, **G** and **H** = Late embryogenesis abundant (LEA) proteins, **I** and **J** = WRKY DNA, **K** and **L** = Heat shock proteins, **M** and **N** = Chaperones and **O** and **P** = Transcription factors.

Expression patterns (shown by log2FC) of 55 manually selected defense response genes were examined over the time course (6 hag, 1 dag, 2 dag, 5 dag, 8 dag, 45 dag and 54 dag) in Albert and Namikonga (Fig. 6). The genes were manually selected based on earlier studies in which these genes were shown to function in pathogen defense. The genes included those with leucine-rich repeats (LRRs) (Fig. 6A and B), those that have a nucleotide binding domain (NBARC) (Fig. 6C and D), pathogenesis-related (PR) proteins (Fig. 6E and F)^48^, late embryogenesis abundant (LEA) proteins (Fig. 6G and H), transcription factors WRKY; (NAM, ATAF and CUC (NAC)); NmrA; eIF; GATA; and GRAS) (Fig. 6I,J,O,P), chaperones (Fig. 6 K and 6L) and heat shock proteins (HSP) (Fig. 6M and N). Both LRR and NBARC domains are structural units of the NOD-like receptors (NLRs), which are defense proteins whose structure contains a TIR or CC domain at the N-terminus, LRRs at the C-terminus and a centrally located NBARC domain^49^. Of these, seven gene families (LRR, NBARC, PR, LEA, Chaperone, HSP and TF) were significantly different between Albert and Namikonga, and the details are presented below.

#### LRR domain

In Namikonga, two (007501 and 001048) of the five selected LRR genes were up- regulated at 2 dag and four (007501, 000978, 034154 and 000765) were up-regulated at 5 dag. For Albert, only two genes (007501 and 001048) were up-regulated at a later time point, 54 dag.

#### NBARC domain

For Namikonga, all six selected NBARC genes were strongly up-regulated at 5 dag with similar profiles, suggesting their co-expression and co-regulation. For Albert, only a few genes were induced (034172 and 022172) at that same time point.

#### PR proteins (thaumatin-like superfamily, PR-5 family)

Two PR genes (012383 and 011960) that belong to the PR-5 family^50–52^, thaumatin-like superfamily, were analyzed in Namikonga and Albert. In Namikonga, gene 012383 was up-regulated at 2 and 5 dag, while gene 011960 had delayed induction. In Albert, however, gene 012383 was up-regulated at 1 dag and later at 45 dag.

#### LEA proteins

In Namikonga, gene 019959 was highly induced in Namikonga at 2, 5 and 8 dag, while gene 025676 was induced at 1, 2 and 5 dag but at a lower level than that of gene 019959. In Albert, however, both genes remained unchanged across all time points.

#### Chaperones

Of the 14 selected chaperones, gene 026342 was induced in both Namikonga and Albert at 2 dag but to a greater extent in Namikonga (log2FC = 18) compared to Albert (log2FC = 5). The same gene (026342) was repressed in both varieties at 45 dag. In Namikonga, several other chaperones were induced at 2 and 5 dag; however, gene 026342 showed the only interesting pattern in Albert, with the other genes remaining unchanged.

#### HSPs

In Namikonga, six (014648, 009750, 010803, 033681, 034243 and 003240) of the 14 selected HSPs were highly induced at 2 dag (log2FC = 3.6–7.5). The other eight genes were also induced at other early time points (6 hag, 1 dag, 2 dag and 5 dag) in Namikonga. For Albert, only two genes, 010803 (induced at 6 hag, 1 dag and 2 dag) and 003340 (induced at 45 dag), were interesting. The others remained unchanged.

#### TFs (WRKY, NmrA, GATA, GRAS and NAC)

Of the five families of TFs examined, only two showed interesting patterns. The first, WRKY, had two genes (011680 and 014614) that were both repressed at 2 dag and induced at 5 and 8 dag in Namikonga, whereas only 011680 was induced in Albert at 8 dag. The other TF families (NmrA, GATA, GRAS and NAC) were induced at 2 and 5 dag in Namikonga only. In Albert, two genes (032524 and 028589) that encode NmrA proteins were induced at 45 dag, while the others remained unchanged at all other time points.

**Other defense-related genes identified in an earlier study by Maruthi** *et al*.^20^: A transcriptome analysis of Kaleso (identical to Namikonga^53^) and Albert infected with CBSV reported that three NAC transcription factors (011029, 015961 and 023870) and one elongation factor, eIF(iso)4E (016601) were over-expressed. The NAC transcription factors were over-expressed in CBSV-infected Kaleso by 27–139 times compared to CBSV-infected Albert^20^, while the eIF was two-fold over-expressed in CBSV-infected Kaleso compared to Albert, in RPKM values. We examined the fold change of the count values of these genes in the present study (Supplementary Tables S6 and S7). Preliminary filtering dropped one NAC gene (016601) together with other genes whose expression was below the expression threshold (see the details of filtering low-quality genes in the methodology section “Filtering RNAseq reads”). The remaining two NAC genes were expressed as follows: In Namikonga, both NAC genes (011029 and 015961) were up-regulated at 2 dag (log2FC of 1.0 and 2.9) and 5 dag (log2FC of 5.1 and 1.0). In Albert, both genes were slightly up-regulated (log2FC of 0.4) at 2 dag. At 5 dag, one NAC gene was substantially down-regulated in Albert (log2FC = −2.4), and the other remained slightly up-regulated (log2FC = 0.4).

## Discussion

CBSD is a major constraint to the production of the African staple food, cassava^1^. Two causative agents have been identified, the Potyviruses CBSV and UCBSV. There is an urgent need to identify molecular markers or biomarkers associated with resistance or tolerance to support the efficient breeding of new cassava varieties. Here, we used an RNAseq approach to identify DEGs between UCBSV-inoculated and mock-inoculated plants in a time-course experiment with two Tanzanian cassava varieties, Namikonga (CBSD resistant) and Albert (CBSD susceptible). The results indicated that Namikonga plants restricted disease progression, limited symptoms to the leaves and maintained a low virus load, while allowing normal root expansion (root bulking) without necrosis or constriction. In Albert, where viral loads were higher, infection with UCBSV caused substantial leaf chlorosis and root necrosis. The results also indicated that a strong resistance response is invoked in Namikonga from approximately 2 dag to at least 5 dag, reducing by 8 dag. Comparatively little differential gene expression was observed at the early time points (6 hag to 1 dag). The over-represented GO terms indicate the involvement of ‘translation initiation’ in the ‘ribosomal subunit’ and ‘phosphorelay signal transduction and response regulation’. In addition, many genes with defense-related roles, including those with LRR domains, NLRs, PR proteins, LEA proteins and TFs are highly differentially expressed at these time points, indicating a complex response. Although some of these genes are up-regulated in Albert, significant up-regulation tends to occur much later, at 54 dag.

### Low virus titer in Namikonga suggests that the defense mechanism controls virus replication

Typical foliar CBSD symptoms were observed on both Namikonga and Albert plants at the late sa﻿mpling phase﻿ ﻿(﻿﻿54﻿ dag﻿), and a﻿t harvest, demonstrating that Namikonga is not immune to UCBSV and can be graft inoculated (Fig. 1). UCBSV-inoculated plants of both varieties tested positive for UCBSV (Fig. 1) using CBSDDR/CBSDDF2 primers^54^. No UCBSV sequences were identified in the Namikonga RNAseq reads, except at 45 and 54 dag. The high number of UCBSV sequences recovered from Albert at the early time points confirms that the virus multiplied at a higher rate in Albert than in Namikonga (Fig. 3). Susceptible cassava varieties have been shown to harbor a higher virus load than resistant varieties^21, 55^. Low UCBSV titers have been reported in field samples of Namikonga that were naturally infected via the (U)CBSV vector, the whitefly^21, 55^. We observed similar trends, as the UCBSV titer remained very low throughout the study and only increased at 54 dag, whereas that of Albert had doubled by 6 hag compared to virus levels in Namikonga at the same time. The slightly increased titer (117 UCBSV reads) at 54 dag in Namikonga might be a result of localized virus multiplication within the leaves of Namikonga, which was previously observed in another study^20^, or may indicate the occurrence of a more general fluctuation in viral load, rather than loads remaining constantly low. There was an exponential increase in virus load in Albert at 1 dag, which reduced by 5 dag. Altogether, virus levels remained higher in Albert than in Namikonga up to 54 dag (1660 UCBSV reads), except at 5 dag, when both varieties had very low loads. This observation suggests that defense mechanism in Namikonga controls virus multiplication without necessarily eliminating the virus. Our data support earlier studies in which Namikonga was declared ‘resistant’ to CBSD^20^.

### Read mapping to the cassava reference genome

Over 75% of the raw reads mapped to genic regions of the cassava reference genome. Of the unmapped reads, 2% mapped to multiple gene models, irrespective of length. Other possible explanations for the unmapped reads could include sequence variation from SNPs between the cassava reference genome version and Namikonga and Albert. The cassava reference genome is from a partial inbred line (accession number AM560-2) derived by three generations of selfing from variety MCOL-1505 of Latin American origin^42^, while Namikonga is a local variety derived from early cassava breeding work at Amani, Tanzania^56^, and is known to contain approximately 14% *M*. *glaziovii* – *M*. *esculenta* hybrid genome^57^. Albert is a local, pure *M*. *esculenta* variety from Tanzania^57^. These differences could explain the unmapped reads, as population structure does exist between cassava germplasms from South America and Africa^58^.

### GO term enrichment of DEGs

The most over-represented GO terms were identified at 2 dag and 5 dag in Namikonga, which corresponded to the largest number of DEGs (3887 and 4911, respectively, in Namikonga, compared to two and zero, respectively, in Albert). Of the enriched GO terms at these time points, those involved in plant defense included ‘translation elongation factors’ (containing eIF genes), ‘ribosomal subunit’ and ‘phosphorelay signal transduction’. None of these GO terms were over-represented in Albert. This observation suggests that translation elongation, the ribosomal subunit and phosphorelay signal transduction have play key roles in the resistance of Namikonga to CBSD.

#### Translation elongation is among the defense responses in Namikonga

Some viruses utilize the host plant’s translation factors to replicate within the host. The virus-encoded protein cap structure (VPg) covalently linked to the 5′ end of some viral genomes, including (U)CBSV, recognizes and binds to translation eIFs on ribosomal subunits. This delivers an RNA helicase to the 5′ region, bridges the mRNA to the ribosome and circularizes the mRNA, enabling viral replication^33, 59^. Mutations in translation initiation factors can result in structural changes in the protein, likely preventing the interaction of viral RNAs or proteins with host factors and therefore restricting the multiplication or movement of the virus, indirectly rendering the host resistant to that virus. The involvement of eIF genes in passive host plant resistance through an altered virus-host interaction surface has been recognized for many years. Two of the eIFs most commonly implicated in passive host plant resistance are eIF4E and eIF4G and their isoforms eIFiso4E and eIFiso4G. This passive resistance has been widely demonstrated in Potyviruses (reviewed in refs 28, 33 and 60), the family to which UCBSV belongs^61^. eIF4E has been repeatedly identified as a naturally occurring, recessively inherited resistance locus in species such as *Pisum sativum* (*sbm1*, *wlv/cyv2*)^62^ and *Solanum habrochaites* (*pot-1*)^31^. Alleles *pvr1* and *pvr1* ^2^ from *Capsicum spp*.^32^ confer resistance to the potyvirids TEV, PVY and PMV based on eIF4E. Similarly, over-expression of *pvr1* from *Capsicum* spp. confers resistance to TEV and PMV in *Solanum lycopersicum* based on eIF4E, and over-expression of *pvr1* ^2^ confers resistance to PVY in *Solanum tuberosum*. The resistance gene *pvr6*, which functions with *pvr1* or *pvr1* ^2^, confers resistance to PVMV and ChiVMV in *Capsicum* spp. based on eIF(iso)4E. In Arabidopsis, an elF4E gene confers resistance to melon necrotic spot virus (MNSV)^30^. Over-representation of the ‘translation initiation factor’ and ‘ribosomal subunit’ GO terms in Namikonga at 2 dag and 5 dag (Table 2) indicates the presence of an eIF-mediated resistance mechanism in the Namikonga defense response. In addition to these, the term ‘phosphorelay signal transduction’ was also enriched at 2 dag and 5 dag in Namikonga.

#### Phosphorelay signal transduction activates the defense response in Namikonga

The phosphorelay system is a complex interaction of multicomponent regulatory systems in which pathogen-triggered signals are transferred from host extracellular histidine- or histidine-containing proteins to intracellular regulatory proteins, which in turn trigger reactions that alter the plant’s physiological patterns of defense^63–67^.

More specifically, a phosphoryl group is transferred from a membrane-bound, conserved, sensor histidine kinase to an intracellular, receiver response regulator containing a conserved aspartate residue. The histidine kinases receive signals from extracellular sensors and transmit these signals via multi-step processes to cytoplasmic response regulators. The response regulators localize in the nucleus alone or in both the nucleus and the cytoplasm. The transfer of stimuli between the extracellular histidine kinases and intracellular response regulators occurs in a multi-step manner involving several plant proteins^63, 64^. The response regulators in turn trigger a cascade of reactions that lead to pathogen defense and other physiological pathways.

Cytokinins, which are plant growth hormones, have been shown to receive and transmit signals in a manner similar to that of bacterial two-component phosphorelay signaling. In Arabidopsis, cytokinins have receptors (AHK2, AHK3 and AHK4)^68^ whose role is similar to that of eukaryotic histidine kinases in the bacterial two-component system. These three proteins have extracellular cytokinin binding, cytoplasmic histidine transmitter and receiver domains, respectively^65, 69^. Arabidopsis plants treated with cytokinins were resistant to *Pseudomonas syringae* pv. tomato DC3000 (Pst)^70^. Defense against Pst occurs via the salicylic acid (SA) pathway, which is triggered by cytokinins^71^. A receptor-like kinase (*Pseudomonas syringae* effector B) was shown to activate the expression of RPM1 (an NBS-LRR protein) against Pst in Arabidopsis^47^. The SA pathway confers resistance to the Potyviruses PVY in potato^72^ and peanut mottle virus (PeMV)^73^. In tobacco, plants primed with cytokinins became tolerant to the strain PVY^NTN^ of potato virus Y (PVY)^66^. In this study involving cassava and UCBSV, both histidine kinases and regulatory proteins were among the over-expressed genes in Namikonga at 2 dag and 5 dag (Table 4). In addition to these GO terms and the constituent genes, other defense-related genes were significantly expressed in Namikonga compared to Albert.

### A coordinated set of defense genes collectively confer resistance to CBSD in Namikonga

In addition to eIFs and possibly linked to phosphorelay signal transduction, genes that encode LRR- and NBARC-containing proteins, PR proteins, LEA proteins, TFs (WRKY, GRAS, GATA and NmrA), chaperones and HSPs were all highly up-regulated in Namikonga at 2 dag and 5 dag. These proteins have all been implicated in defense responses against viral, fungal and bacterial pathogens. Defense genes encoding proteins containing LRRs (Fig. 6A and B) and NBARCs (Fig. 6C and D) were substantially over-expressed at 2 dag and 5 dag in Namikonga, the latter time point being the peak of expression for most genes (Fig. 6). Other defense genes, including those encoding PR proteins (Fig. 6E and F), LEA proteins (Fig. 6G and H), TFs involved in pathogen defense (WRKY, NmrA, GATA, NAC and GRAS) (Fig. 6E,F, O,P), HSPs (Fig. 6M,N) and chaperones (Fig. 6K,L), were over-expressed at 2 dag and 5 dag. This suggests that Namikonga’s resistance to UCBSV infection may be complex, involving several possibly interrelated strategies including NLR proteins with LRR and NBARC domains.

#### An NBS-LRR network contributes to Namikonga’s resistance to UCBSV

In general, the NBARC genes were over-expressed at 5 dag in Namikonga and steadily decreased thereafter. In Albert, some genes were down-regulated and others over-expressed (but with lower log2FCs than in Namikonga) at 5 dag. However, three (000058, 001752 and 029764) of these genes were over-expressed at 45 dag in Albert. Maximum LRR gene expression was observed in Namikonga at 2 dag and 5 dag. When the LRR genes were maximally expressed in Namikonga, their expression was either normal or down-regulated in Albert. Two of these genes (001048 and 007501) were over-expressed at a much later time point (45 dag) in Albert, a time point when the expression of these genes in Namikonga had reduced to near normal.

Typically, LRRs, NBARCs and CCs or TIRs are sub-domains of the tri-modular NLR protein, the most studied type of defense gene^49, 74, 75^. Alone, LRR and NBARC domains can function in pathogen defense. Some NLR proteins require the presence of other ‘helper’ proteins to develop a resistance phenotype. The ‘helper’ is often an HSP^76, 77^. In the case of Namikonga, synchronized timing for the up-regulation of these HSPs, chaperones and NLRs implies a synergistic interaction of the three defense proteins to orchestrate Namikonga’s resistance to CBSD.

HSPs are structurally disordered proteins with diverse functions in regulatory, signaling and defense pathways. Alone, HSPs may function as defense proteins against biotic and abiotic stresses^78, 79^. Some HSPs are also chaperones. Chaperones are mainly involved in RNA binding and protein folding^80, 81^, a role that is key in the functioning of elongation factors^46^. Their potential ability to independently confer resistance phenotypes in Namikonga cannot be ruled out.

During the peak over-expression of LRR, NBARC and HSP genes, LEA proteins were also over-expressed, reaching a maximum at 8 dag in Namikonga (Fig. 6H). LEA proteins are involved in adaptation to water stress^82^ and are abundant in plant^82, 83^, fungi^84^ and mammalian genomes^85–89^. As the name suggests, LEA proteins are produced during the late stages of embryogenesis, especially at seed development. They protect cells from desiccation^82^, salinity^90^ and extreme cold or hot temperatures^85^ in a dose-dependent manner^88^. The expression of LEA genes in Namikonga seems to ensure that the already infected plants maintain balanced cell solutes for normal functioning under the stressful conditions imposed by UCBSV.

Another type of defense proteins that were up-regulated in Namikonga at 2 dag and 5 dag are the PR proteins. The PR proteins are inducible defense-related proteins of varied molecular sizes (5 to 75 kDa)^91^. They were first discovered in tobacco resistant to infection by TMV^48, 51^. To date, PR proteins have been associated with resistance to several pathogens^48, 51^. The proteins are RNase and DNase active, implying that they function in defense by abolishing foreign RNA and DNA molecules in host plants. The PR proteins have antiviral properties^92–94^, and resistance triggered by PR proteins causes an HR and programmed cell death at the infection site^92, 93^. They are critical for secondary metabolite biosynthesis, storage, phytohormones and ligand binding^94^. Their expression marks the peak of systemic acquired resistance (SAR) against pathogens^51^. In Namikonga, PR proteins were up-regulated at the peak of LRR, NBARC and HSP expression, but the role and extent of involvement of PR proteins in resistance to UCBSV is not clear.

At the same peak time points, five major groups of TFs (NAC, WRKY, GRAS, GATA and NmrA) were over-expressed in Namikonga. Although there is very little knowledge of the transcriptional networks that are activated in cassava plants in response to pathogens, the co-expression of these transcription factors in Namikonga with other defense genes in response to UCBSV inoculation suggests a role in defense.

The NAC (NAM, ATAF and CUC) superfamily of TFs is one of the largest TF families. It is found only in plants and was thus investigated here. The role of NAC proteins in defense is known in several plants. In Arabidopsis, a functional SA pathway is required for NAC-directed defense against viral pathogens such as TCV, but the jasmonic acid (JA) and ethylene (ET) pathways are not essential^95^. A NAC-mediated HR causes resistance in rice to rice blast disease^96^, in maize to *Colletotrichum graminicola* ^97^ and in Arabidopsis to *Botrytis cinerea*, *Pseudomonas syringae* pv. *tomato* (Pst)^98^, *Turnip crinkle virus* ^95^ and *Acidovorax avenae* ^99^. In this study, NAC genes were differentially expressed at specific time points with other defense genes, implying that NAC has a role in defense. However, to date, no studies have focused on the role of NAC genes in cassava’s defense against viral pathogens. Our data indicate that the induction of NAC genes in Namikonga at 2 dag and 5 dag contributes to NAC-mediated resistance to CBSD in Namikonga.

Another class of transcription factors, WRKY, was also up-regulated in Namikonga at 2 dag and 5 dag. The role of WRKY transcription factors in plant defense^100–102^ and their structural features and functional network have been reviewed^103^. In rice, two alleles of a WRKY gene (OsWRKY45-1 and OsWRKY45-2) conferred resistance to *Magnaporthe grisea*, yet each allele encoded a different set of defense genes regulated by distinct promoter regions^104^.

In this study, Namikonga had two differentially expressed WRKY genes at 2 dag and 5 dag. However, one WRKY was down-regulated at 2 dag, possibly to establish a network feedback loop to regulate their over-expression at 5 dag and 8 dag, respectively^104^. In some cases, SA and JA enhance the functionality of WRKY genes in defense. In rice, enhancing the levels of SA and JA production enabled OsWRKY45-1 to resist Xoo and Xoc, but OsWRKY45-2-mediated resistance to Xoo and Xoc required JA only^104^. Whether the SA levels enhanced the activity of the Namikonga WRKY genes against UCBSV was not examined in this study. There is a need to examine the roles of SA and JA in the Namikonga defense mechanism and if and how these phytohormones enhance the action of WRKY genes.

### Concluding remarks

**Figure 7:**
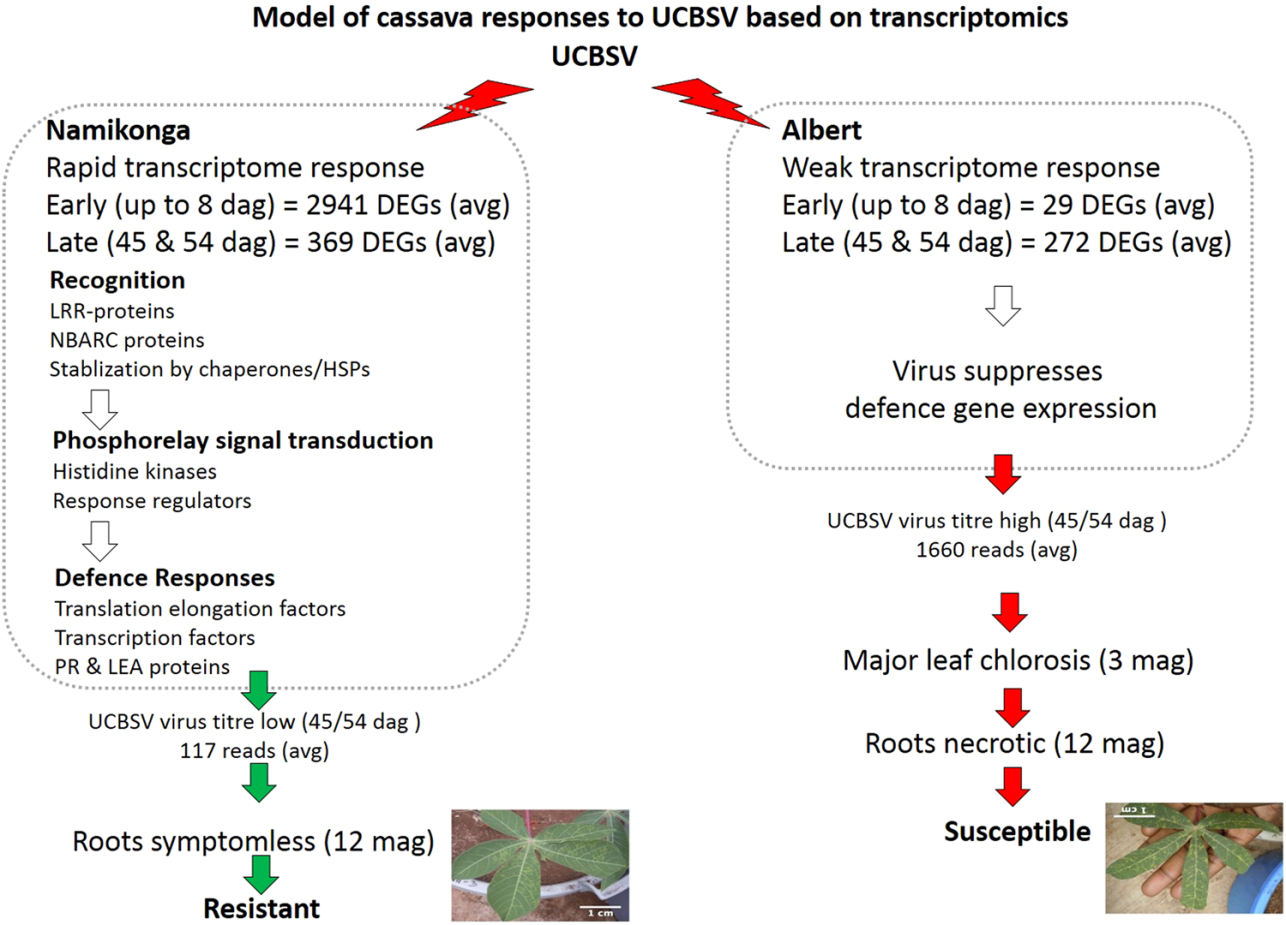
Model of cassava responses to UCBSV based on transcriptomics. DEGs = differentially expressed genes; avg = average; dag = days after grafting and mag = months after grafting.

Based on these findings, a model for resistance to CBSD in Namikonga is proposed (Fig. 7). In this model, the challenge of Namikonga with UCBSV triggers an NBS-LRR signalling cascade, which subsequently induces a phosphorelay signal transduction pathway leading to defence. This is marked by induction of defence-related PR-proteins and stress response LEA, heat-shock and chaperone proteins, and a range of proteins associated with translation, including translation elongation factors. This may represent a mechanism in Namikonga of preventing efficient interaction of UCBSV with the host translational machinery, a known mechanism of resistance to potyviruses in other plants^31, 32, 61, 62, 105^. The outcome is that Namikonga shows reduced virus titre, limited leaf chlorosis and minimal (if any) root necrosis. In Albert, UCBSV’s presence does not induce any recognizable defence pathway and virus titre increases unchecked (Fig. 7). The result is major leaf chlorosis and root necrosis. Our study confirms that resistance in Namikonga is associated with significant transcriptional re-programming soon after UCBSV challenge, in contrast to Albert in which the subterfuge of the virus appears to suppress defence gene expression (Fig. 7). Furthermore, our data provides a rich source of candidate alleles in the Namikonga genotype for testing hypothesis of resistance mechanisms by correlating gene expression with QTL mapping data, with the ultimate aim of developing robust biomarkers for cassava breeders to develop durable resistance to CBSD.

## Methodology

### Planting material sources

The study was conducted on Namikonga, a CBSD-resistant variety, and the susceptible variety Albert^21^. Namikonga is an interspecific hybrid between wild (*Manihot glaziovii* Müll. Arg.) and domesticated (*Manihot esculenta* Crantz) cassava, with an estimated 14% interspecific hybrid genome^57^. Albert is a farmer variety from southern Tanzania; it is susceptible to both virus species, showing characteristic CBSD-associated leaf chlorosis and severe root necrosis^21^. Virus-negative stem cuttings of Namikonga and Albert were obtained from plants maintained in a greenhouse at the Sugarcane Research Institute (SRI), Kibaha, Dar es Salaam, Tanzania. The source of inoculum, variety NDL06/132, was from a field trial at the SRI, Tanzania and was multiplied for scions in a greenhouse at BioSciences eastern and central Africa (BecA), Nairobi, Kenya. Experimental plants were tested for the presence or absence of both CBSV and UCBSV prior to grafting. For this, RNA was extracted from fresh leaf samples using a CTAB protocol, with the amount of starting extraction buffer material modified from 600 µl to 800 µl^106^. Respective second-strand cDNAs were synthesized using Superscript III (Invitrogen, CA). End-point RT-PCR was performed using primers that distinguish both viruses simultaneously; namely, CBSDDR/F2^54^ and CBSVF2/R8^107^.

### Establishment of the experiment, graft inoculation and sampling

The aim of this transcriptome time-series experiment was to determine the transcriptional signatures immediately after virus infection by grafting (early sampling) and several days before aerial symptom emergence (late sampling). Grafting is commonly used to transmit viruses in cassava, and was used here^4, 16^. Artificial infectious clones were not available for inoculation, transmission using whiteflies was unreliable and mechanical transmission of CBSVs is only effective with model plants^15^. Initially 45 cassava cuttings of each variety were planted in large pots containing 3.85 kg gravel (construction grade) topped to 33.23 kg with forest soil and immediately watered with 3 liters of tap water. Thereafter, the plants were given 2 liters of tap water per week until harvest. At two months after planting, plants were ready for top grafting. Just prior to grafting a baseline leaf sample (time zero) was collected from the top leaves of all plants (see methodology below). Fifteen plants of Namikonga and Albert were grafted with scions positive for CBSV only, 10 plants of each variety were grafted with scions positive for UCBSV only and another 10 plants of each variety with scions positive for CBSV + UCBSV. Ten plants of each variety were grafted as controls with virus negative NDL06/132 scions. Samples were taken as described below from all grafted plants, and frozen until selection of biological replicates was made based on RT-PCR diagnostic results. An RT-PCR test at 3 mag (data not shown) confirmed that only one out of 15 grafted Namikonga plants was positive for CBSV, while seven of 10 plants each grafted with UCBSV or CBSV + UCBSV infected scions were positive for the respective virus. In Albert seven of the 15 plants grafted with CBSV infected scions were positive, and seven of 10 plants each infected with UCBSV or CBSV + UCBSV were positive. It is on the basis of successful graft transmission of UCBSV that this species was chosen for downstream studies. Three biological replicates for each of the two treatments (UCBSV-inoculated and mock-inoculated plants) of Albert and Namikonga were selected for RNAseq.

In this time-series experiment, samples were collected from the main experiment at 6 hag, 1 dag (i.e., 24 hag), 2 dag (i.e., 48 hag), 5 dag, 8 dag, 45 dag and 54 dag. The time points up to and including 8 dag constituted the “early sampling” and the remaining samples the “late sampling”. The “late sampling” time points were selected to coincide with the period just before foliar symptom emergence. To determine this a number of indicator plants were grafted three weeks before the main experiment providing ample time to plan for pre-symptom emergence sampling. At each time point leaves were sampled from below the graft point to avoid sampling the leaves of the sprouting NDL06/132 scion. Typically both Albert and Namikonga plants have at least five lobes per leaf, each lobe weighing >400 g. Therefore, we sampled a different lobe of the same leaf at every different time point. Where lobes were fewer than needed to cover all successive time points, samples were taken from lobes on the leaf below in the same order. The samples were placed in pre-labeled aluminum foil envelopes and immediately frozen in liquid nitrogen, transferred to −80 °C and stored prior to RNA extraction. At harvest (12 mag), roots from all plants were scored for CBSD-related necrosis.

#### CBSD symptom scoring

UCBSV-inoculated and mock-inoculated plants were scored for above-ground symptoms ﻿during the late sampling phase (up to 54 dag)﻿﻿, and additionally﻿ at harvest (12 mag) for symptoms in storage roots, according to standard 1–5 scales^108^. According to the leaf severity scale, 1 implies no symptoms, and 5 implies clear leaf chlorosis covering >75% of leaves and streaking on the stem with shoot die back. On the storage root severity scale, a score of 5 implies more than 50% root necrosis^7^.

### RNA extraction, cDNA synthesis and RNAseq

Frozen leaf samples were ground to a fine powder in liquid nitrogen and divided into three aliquots for (a) cDNA library synthesis, (b) virus diagnostics using RT-PCR and (c) a back-up. RNA was extracted using the Spectrum Plant Total RNA kit (Sigma-Aldrich) following the manufacturer’s protocol. RNA integrity was confirmed using a Qubit fluorometer (Life Technologies). The RNA extraction, cDNA library synthesis, shipment and Illumina sequencing were performed 16 months apart for two batches of samples that were sequenced at two facilities, the University of California, Berkeley, CA, USA, and Dow AgroSciences, Indianapolis, IN, USA, due to funding availability. The same person in the same laboratory at BecA conducted the RNA extraction and cDNA library synthesis of both batches. Each batch contained both the mock-inoculated and UCBSV-inoculated samples from a genotype at a specific time point, as listed in Supplementary Table S1. The same shipping company was contracted to ship each batch of cDNA libraries to the sequencing facilities. The cDNA libraries were synthesized using the Illumina Truseq cDNA library synthesis kit (Illumina, San Diego, CA, USA) using set A indices for 50-bp sequencing at UC Berkeley and set B indices for 101-bp sequencing at Dow AgroSciences. Illumina Hi-Seq instruments were employed at both facilities, using in-house pre-sequencing preparations of cDNA library material and instrument run settings with the aim of generating 20 million reads of 50 bp (for UC Berkeley) or 101 bp (for Dow AgroSciences) in length for each biological replicate sample (Supplementary Table S1). Control and UCBSV-infected samples from both varieties obtained at 6 hag and 8 dag were sequenced at UC Berkeley. Control and infected Albert samples collected at 45 dag and 54 dag were also sequenced at UC Berkeley. For both varieties, control and UCBSV-infected samples collected at 1 dag, 2 dag and 5 dag, were sequenced at Dow AgroSciences. Dow AgroSciences also sequenced the Namikonga samples (mock- and UCBSV- inoculated) collected at 45 dag and 54 dag.

### RNA extraction and cDNA synthesis for diagnostics

For RT-PCR virus diagnostics, aliquots of RNA samples from the Spectrum extractions described above were used, and cDNA was synthesized using Superscript III (Invitrogen). The presence of UCBSV was positively verified with a single band at 440 bp, and the absence of CBSV was verified using the primer sets described above with appropriate CBSV and UCBSV positive and negative controls^54^ (Fig. 2).

### Detection of UCBSV in RNAseq reads

The availability of RNAseq data from this experiment was exploited as an additional method to detect UCBSV RNA virus molecules in the cassava samples. To confirm successful graft inoculation with UCBSV and that control samples were UCBSV-negative, RNAseq reads from all 96 samples were mined for UCBSV sequences. To retrieve virus sequences from RNAseq reads, all five fully sequenced UCBSV genomes from NCBI were used as a reference to map the RNAseq reads of UCBSV-infected and mock-inoculated samples. The five UCBSV genomes were isolates HG965222.1, FN434109.1, FJ185044.1, HM181930.1 and NC_014791.1 and were combined to form one UCBSV reference file. RNAseq reads that failed to map to the cassava reference genome (unmapped reads) were each aligned to the UCBSV reference genome using Bowtie2^41^. The results were obtained from the alignment summary and transferred to Excel for plotting.

### Data analysis

#### Read quality analysis and mapping to the cassava reference genome

Individual files received as batches of ≤four million reads (later combined to make one large file of 20 million reads) were analyzed for sequence quality using FastQC (FastQC)^109^. All reads had a Phred score above 20, meaning that 99% of the bases were accurately called. The first low-quality 10–13 bp of each read, which is typical for Illumina sequencing, were trimmed to 37 bp (for 50-bp reads sequenced at UC Berkeley) and 88 bp (for 101-bp reads sequenced at Dow AgroSciences) using FASTX_toolkit (fastx_trimmer)^110^. The trimmed files were mapped to the cassava reference genome v4.1 (plant accession number AM560-2)^42^ using Tophat2^41^, which runs Bowtie2 in the background. The default settings were used, allowing up to two mismatches per read. Allowing two mismatches for both 37 bp and 88 bp reads did not affect mapping, as reads that map to the same gene model are only counted once, irrespective of their length, taking into account the two allowed mismatches. This was tested and confirmed using randomly selected samples of 37 bp or 88 bp (data not shown). The mapped reads were de-duplicated using Picard’s dedup function, correcting for any amplification bias caused during the PCR used in constructing the sequencing libraries. Mapped reads with gene models on the cassava genome were counted with HTseq_count^43^, a python script widely used to count RNAseq reads^43^. Genes without gene models (counted altogether as no_feature) or matching more than one gene model (ambiguous) were removed before clustering and identification of DEGs. The HTseq_count command *no_feature* counts reads that are not aligned to any gene model on the reference genome, while the command *ambiguous* counts reads spanning an intersection of two gene models or aligned to more than one gene model (Supplementary Data S4).

#### Filtering RNAseq reads

To reduce artifacts and increase the statistical power for identifying DEGs, the counted reads were filtered in four steps. First, genes with zero count values were deleted, followed, second, by genes with very few reads (rowsum ≤log_2_5) on the distribution curve (data not shown). Third, genes that passed stages (i) and (ii) but were below a set per time point minimum cut-off were deleted. The minimum cut-off per time point was calculated from row (per gene) median and standard deviation values (minimum cut-off = median − 2 * standard deviation). The fourth and last filtering was based on how close the biological replicates clustered by respective treatments, variety and time point. Outlier biological replicates were removed from the downstream analysis, although at least two samples per treatment were retained. As was done for differential gene expression analysis, data for clustering were normalized using the *estimateSizeFactors* function of the DESeq package^45^. Data normalization was performed with combined gene counts from both Albert and Namikonga. To do this, only good-quality genes (genes that passed the four filtering stages described above) were used for the normalization analysis in both varieties. Clustering was performed using Pearson’s algorithm. The algorithm clusters samples based on their covariance, providing a robust method of grouping samples as opposed to the distance-based Euclidean method. The filtering, clustering, selection of DEGs and plotting of data were performed using the R statistical package^111^.

#### Identification of DEGs and cluster analysis

Compared to distribution-based methods and cuffdiff^112^, negative binomial algorithms implemented by DESeq (edgeR and bayseq) provide a higher statistical power for identifying DEGs^43, 113^. Using filtered reads, DESeq was applied to identify DEGs between control and treated samples of each variety and at each time point. In DESeq, each variety had a dataset for respective sampling time points (example: Albert_6hag). Each dataset contained filtered biological replicates for one time point and two treatments (UCBSV inoculated and mock inoculated). The function *newCountDataSet*, which imports gene counts with pre-defined conditions, was used to import samples under “infected” or “control”, where “infected” identifies gene counts of UCBSV-inoculated samples and “control” identifies gene counts from mock-inoculated samples. Once imported, the samples were normalized by their respective library sizes. To perform ‘normalization’, the effective library size was determined using the *estimateSizeFactors* command. The per sample dispersion was then estimated using the *estimateDispersions* command, followed by differential expression analysis. Differential expression analyses were performed using the negative binomial algorithm with the command *nbinomTest*. After differential expression analysis, p-values were adjusted for multiple testing with the Benjamini-Hochberg procedure, which controls for the false discovery rate (FDR). For most RNAseq studies, a 10% FDR is the recommended limit for identifying significant DEGs^44, 113^. We chose only to apply a FDR of 0.1 as the main filter to identify DEGs, and did not apply a further filter of Log2 fold change. Therefore genes for a treatment comparison with log2FC > 0 and FDR < 0.1 were classified as up-regulated, whereas those with log2FC < 0 and FDR < 0.1 were down-regulated. This was done to capture the maximum number of genes with differential expression across the time points.

#### Functional annotation, GO enrichment and network analysis of DEGs

The lists of DEGs were queried against the cassava genome database at VirtualPlant (http://www.virtualplant.org)^114^ to determine functional annotations and GO enrichment. VirtualPlant is annotated using cassavaCyc data (functional categories) from Phytozome’s version 4.1 of the cassava reference genome (plant accession number AM560-2) (26). VirtualPlant contains 30,666 cassava genes, 28,610 of which have predicted Arabidopsis orthologues that have protein-coding annotations. Thus, the functional annotations are primarily metabolic, as available in cassavaCyc (http://www.plantcyc.org/). The GO terms for cassava genes in VirtualPlant are allocated based on the GO terms of their corresponding predicted Arabidopsis orthologues in TAIR (www.Arabidopsis.org). VirtualPlant therefore harbors GO terms for 10,902, 15,368, and 4,546 cassava genes in the biological process, molecular function and cellular compartment categories, respectively. The manual analysis of defense genes focused on the time points with the highest number of DEGs.

## Electronic supplementary material

Supplementary Data S1

Supplementary Data S2

Supplementary Data S3

Supplementary Data S4

Supplementary Data S5

Supplementary figures and tables
