# Supplementary material for: A time series transcriptome analysis of cassava (Manihot esculenta Crantz) varieties challenged with Ugandan cassava brown streak virus
Source: Sci Rep. 2017 Aug 29;7:9747. doi: 10.1038/s41598-017-09617-z (PMC5575035; doi:10.1038/s41598-017-09617-z)
Supplement: Supplementary file 6 — Supplementary figures and tables [file 41598_2017_9617_MOESM6_ESM.pdf]

A time series transcriptome analysis of cassava (*Manihot esculenta* Crantz) varieties challenged with Ugandan cassava brown streak virus

T. Amuge[1, 2, 3], D. K. Berger[2], M. S. Katari[4], A. A. Myburg[5], S. L. Goldman[4] and M. E. Ferguson[3*]

[1]National Crops Resources Research Institute (NaCRRI), Uganda; [2]Department of Plant and Soil Sciences, Forestry and Agricultural Biotechnology Institute (FABI), University of Pretoria, South Africa; [3]International Institute of Tropical Agriculture (IITA), Kenya, [4]Center for Genomics and Systems Biology, New York University, New York, USA, [5]Genetics Department, Forestry and Agricultural Biotechnology Institute (FABI), University of Pretoria, South Africa

*Corresponding author: M.Ferguson@cgiar.org

Supplementary Figure S1: Clustering of samples based on filtered and normalized RNAseq data, using Pearson's correlation model. Abbreviations: a=Albert, I=infected, h= hours, C=control, n=Namikonga.

Supplementary Figure S1a: Albert

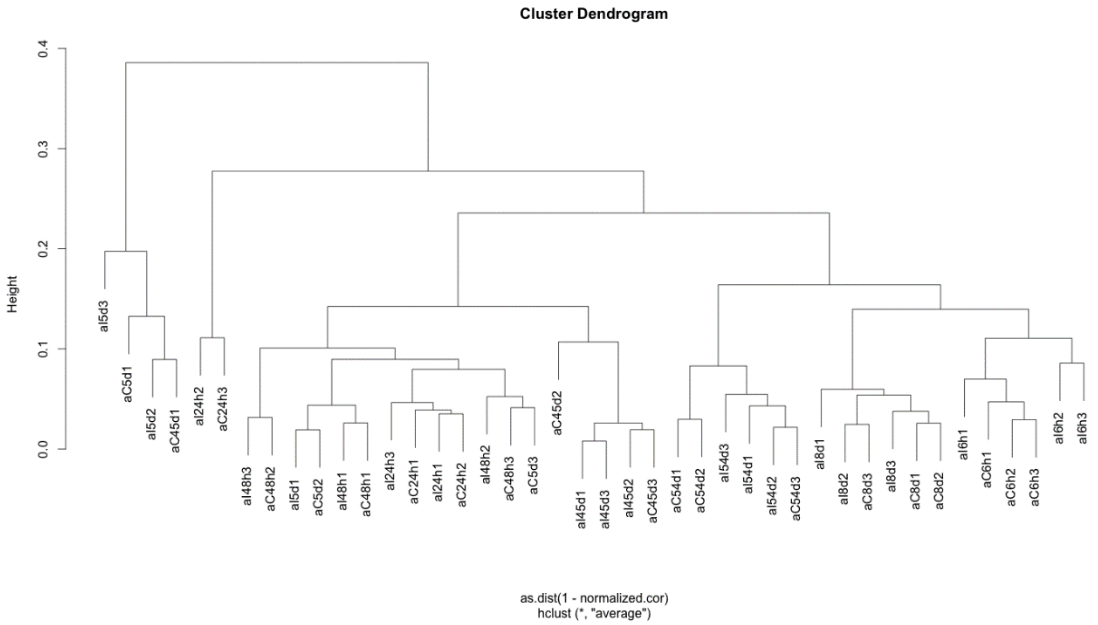

Supplementary Figure S1b: Namikonga

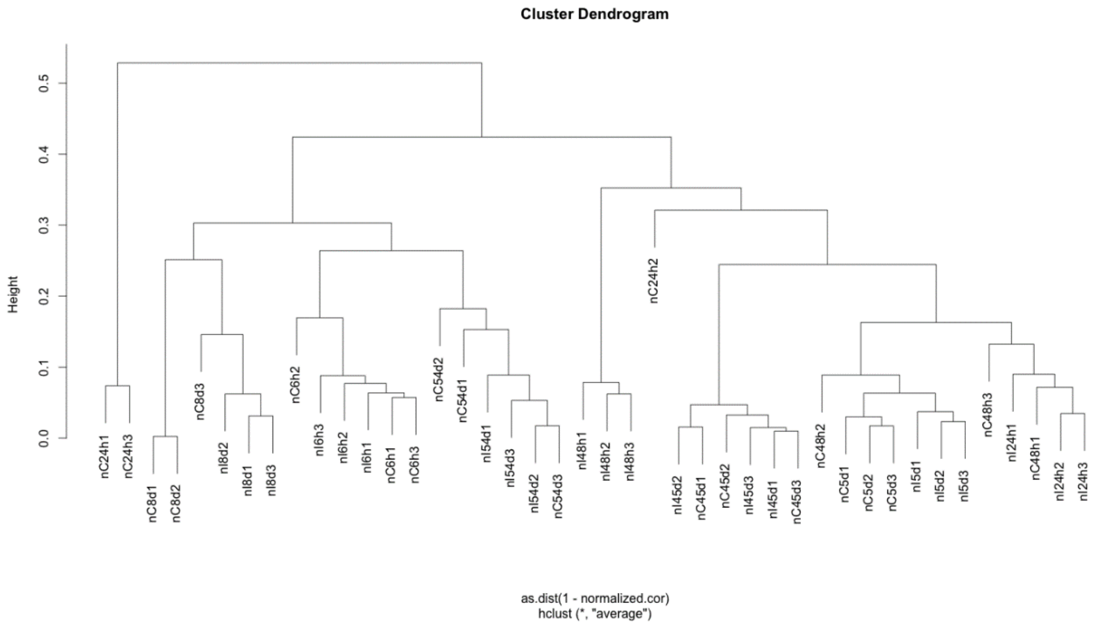

Supplementary Figure S2: Distribution of DESeq normalized reads for Albert (a) and Namikonga (b) after filtering, plotted using box plots. (Sample abbreviations: a=Albert, I=UCBSV-inoculated, h= hours, C=mock-inoculated, n=Namikonga, numbers 1, 2, 3 represent biological replicates 1, 2 and 3 respectively). Samples were taken at seven time points: 6 hours after grafting (6 hag), one day after grafting (1 dag), 2 dag, 5 dag, 8 dag, 45 dag and 54 dag.

Supplementary Figure S2a: Albert

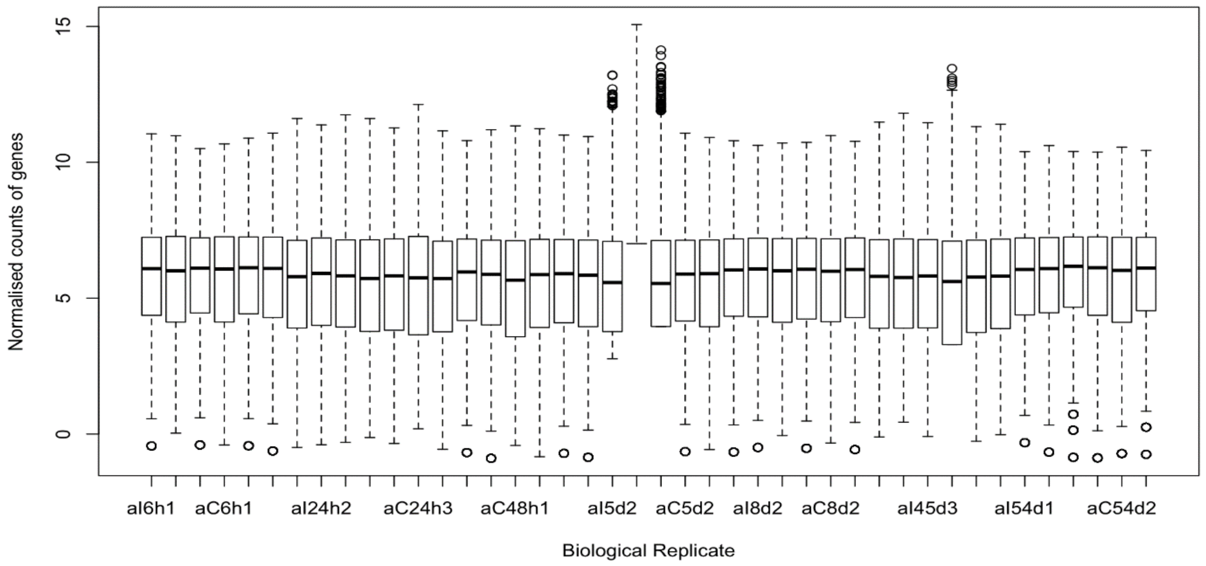

Supplementary Figure S2b: Namikonga

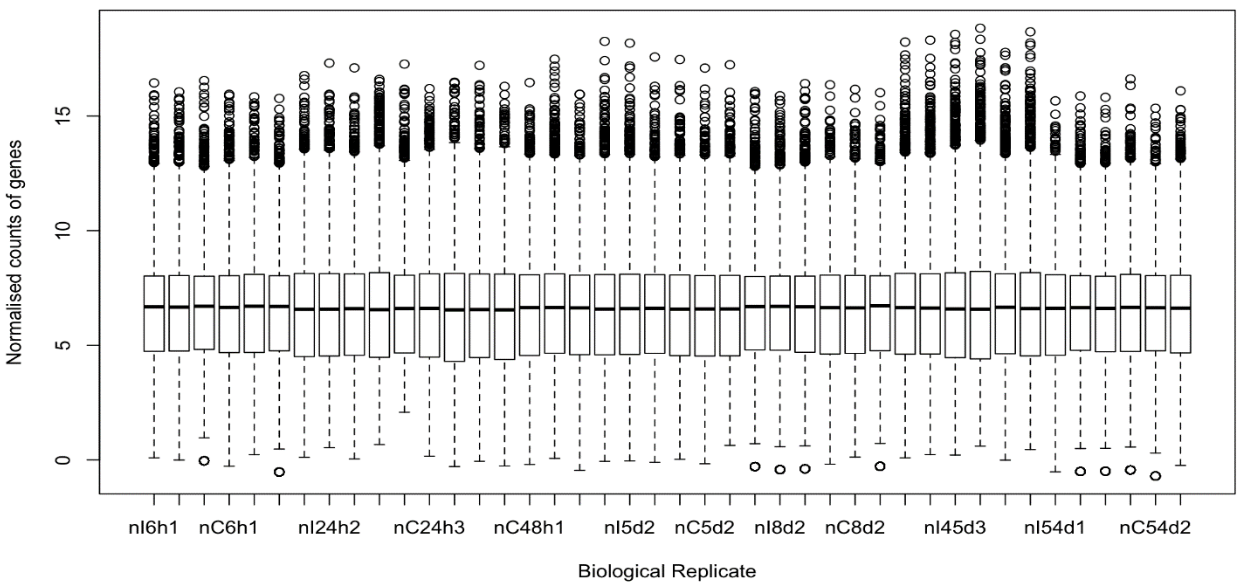

Supplementary Table S1: Laboratories where RNA sequencing was carried out. UCB=University of California, Berkeley; DOW-Dow Agrosciences, Indianapolis

Time point	Albert	Namikonga
6 hag	UCB	UCB
1 dag	DOW	DOW
2 dag	DOW	DOW
5 dag	DOW	DOW
8 dag	UCB	UCB
45 dag	UCB	DOW
54 dag	UCB	DOW

Supplementary Table S2: Numbers of RNAseq reads that were mapped or unmapped to the cassava reference genome v4.1 (plant accession number AM560-2) compared between read-lengths and genotypes.

(a) Output with 50 bp read length

Variety	Mapped	Unmapped	Total Coverage	Mapped (%)	Unmapped (%)
Albert	19,744,151,034	4,754,316,730	24,498,467,764	81	19
Namikonga	11,793,050,691	2,760,319,897	14,553,370,588	81	19
**Total**	**31,537,201,725**	**7,514,636,627**	39,051,838,352		

(b) Output with 101 bp read length

Variety	Mapped	Unmapped	Total Coverage	Mapped (%)	Unmapped (%)
Albert	10,696,774,139	3,477,542,903	14,174,317,042	75	25
Namikonga	20,697,282,109	5,717,006,828	26,414,288,937	78	22
**Total**	**31,394,056,248**	**9,194,549,731**	**40,588,605,979**		

Mapped and unmapped reads were equally distributed in both varieties with 50 bp mRNA reads (a) but Namikonga mapped slightly better (78%) than Albert (75%) with 101 bp reads (b). Altogether, shorter reads mapped better for both varieties (a).

Supplementary Table S3: Number of biological replicates for each cassava treatment used to identify differentially expressed genes using DESeq software[1].

Time-point	Albert		Namikonga	
	**UCBSV-inoculated**	**Mock-inoculated**	**UCBSV-inoculated**	**Mock-inoculated**
6 hag	3	3	3	2
1 dag	2	2	3	3
2 dag	3	2	3	2
5 dag	2	2	3	3
8 dag	3	2	3	3
45 dag	3	2	3	3
54 dag	3	3	3	2

Reference

1 Anders, S., Pyl, P. T. & Huber, W. HTSeq—a Python framework to work with high-throughput sequencing data. *Bioinformatics* **31**, 166-169, doi:10.1093/bioinformatics/btu638 (2015).

Supplementary Table S4: Number of enriched GO terms from up-regulated and down-regulated genes between UCBSV-inoculated and mock- inoculated cassava (varieties Albert and Namikonga) at each time point.

Time point	Albert						Namikonga					
	UP-regulated genes			DOWN-regulated genes			UP-regulated genes			DOWN-regulated genes		
	**B**	**M**	**C**	**B**	**M**	**C**	**B**	**M**	**C**	**B**	**M**	**C**
6 hag	6	2	0	34	10	0	1	9	7	0	0	0
1 dag	0	0	0	3	5	0	29	0	1	7	7	2
2 dag	0	7	0	-	-	-	32	2	0	0	11	1
5 dag	-	-	-	-	-	-	18	6	14	0	0	3
8 dag	-	-	-	0	0	0	46	24	2	23	8	5
45 dag	0	2	4	16	6	6	-	-	-	-	-	-
54 dag	-	-	-	-	-	-	44	22	0	7	36	6

GO enrichment is reported for the following categories: biological process (B), molecular function (M) and cellular component (C) identified using the *biomaps* tool of VirtualPlant 1.3 [1] queried on the cassava genome v4.1 [2]. At some time points, there were no differentially expressed genes (represented by -) or there were no GO terms associated with any of the differentially expressed genes (represented by 0).

References

1 Katari, M. S. *et al.* VirtualPlant: a software platform to support systems biology research. *Plant physiology* **152**, 500-515, doi:10.1104/pp.109.147025 (2010).

2 Prochnik, S. *et al.* The Cassava Genome: Current Progress, Future Directions. *Tropical Plant Biology* **5**, 88-94, doi:10.1007/s12042-011-9088-z (2012).

Supplementary Table S5: Log2Foldchange and adjusted (FDR-corrected) P- values of two eIF4E genes in Albert and Namikonga at all sampled time points.

Time point	Albert				Namikonga			
	G016620m.g		G013223m.g		G016620m.g		G013223m.g	
	Log 2FC	P-adj	Log2FC	P-adj	Log2FC	P-adj	Log2FC	P-adj
	-			1		1		1
6 hag	0.11	1	-0.17		0.30		0.22	
1 dag	0.19	1	0.23	1	-0.13	1	0.01	1
2 dag	0.23	1	0.10	1	-0.19	0.860	-0.17	0.909
5 dag	0.44	1	0.41	1	0.72	0.020	0.63	0.062
8 dag	0.00	1	0.04	1	-0.19	1	-0.26	0.973
	-	1		1		1		
45 dag	0.22		0.17		0.04		-0.07	1
54 dag	NA	NA	NA	NA	NA	NA	NA	NA

Log2foldchange (Log2FC) and level of significance (Adj p-values) of selected cassava4.1_016620m.g (G016620m.g) and cassava4.1_013223m.g (G013223m.g) genes in Albert and Namikonga at all sampled time points.

Supplementary Table S6: Expression ratios in cassava variety Namikonga of 55 manually selected genes that belong to defense-related gene families and that showed significant differential expression in at least one treatment. Gene families included those encoding proteins with Leucine Rich Repeat motifs (LRR), NBARC-motifs, pathogenesis- related (PR) proteins, late embryogenesis abundant (LEA) proteins, heat shock proteins, chaperone proteins, elongation factors (eIF) and several transcription factors (WRKY, NAC, GATA, GRAS, NmrA).

**Gene ID**	**Gene annotation based VirtualPlant***	**6 hag**		**1 dag**		**2 dag**		**5 dag**		**8 dag**		**45 dag**		**54 dag**	
		**Log2 FC**	**Adj. Pvalue**	**Log2 FC**	**Adj. Pvalue**	**Log2 FC**	**Adj. Pvalue**	**Log2 FC**	**Adj. Pvalue**	**Log2 FC**	**Adj. Pvalue**	**Log2 FC**	**Adj. Pvalue**	**Log2 FC**	**Adj. Pvalue**
**LRR**															
Cassava4.1_001048m.g	Leucine-rich repeat transmembrane protein kinase	0.1	>0.05	-0.4	>0.05	1.9	>0.05	-1.3	1.20E-05	0.2	>0.05	-0.5	>0.05	-2.3	>0.05
Cassava4.1_034154m.g	Leucine-rich repeat (LRR) family protein	0.0	>0.05	-0.2	>0.05	1.0	>0.05	1.8	2.24E-07	-0.1	>0.05	-0.2	>0.05	0.7	>0.05
Cassava4.1_007501m.g	Leucine-rich repeat (LRR) family protein	0.4	>0.05	1.2	>0.05	3.8	>0.05	2.6	7.26E-16	-0.1	>0.05	0.0	>0.05	0.2	>0.05
Cassava4.1_000978m.g	Leucine-rich receptor-like protein kinase family protein	1.0	>0.05	-0.1	>0.05	0.3	>0.05	1.5	2.08E-04	0.4	>0.05	-0.2	>0.05	0.4	>0.05
Cassava4.1_000765m.g	Leucine-rich repeat transmembrane protein kinase	0.7	>0.05	1.0	>0.05	0.8	>0.05	2.1	1.36E-02	1.1	>0.05	-0.7	>0.05	-0.1	>0.05
**NBARC**															
Cassava4.1_029764m.g	NB-ARC domain-containing disease resistance protein	-0.1	>0.05	-0.4	>0.05	0.6	>0.05	1.0	1.20E-03	0.2	>0.05	0.2	>0.05	-0.6	>0.05
Cassava4.1_022732m.g	NB-ARC domain-containing disease resistance protein	0.3	>0.05	-0.2	>0.05	0.1	>0.05	1.0	1.43E-03	0.4	>0.05	-0.1	>0.05	-0.8	>0.05

Cassava4.1_022172m.g	NB-ARC domain-containing disease resistance protein	-0.1	>0.05	-0.1	>0.05	0.7	>0.05	1.1	2.33E-03	0.1	>0.05	0.0	>0.05	-0.7	>0.05
Cassava4.1_001752m.g	NB-ARC domain-containing disease resistance protein	0.1	>0.05	-0.1	>0.05	0.6	>0.05	1.1	4.72E-02	0.1	>0.05	-0.4	>0.05	-1.0	>0.05
Cassava4.1_034172m.g	NB-ARC domain-containing disease resistance protein	0.4	>0.05	-0.4	>0.05	0.2	>0.05	1.4	3.68E-02	0.2	>0.05	0.0	>0.05	-1.0	>0.05
Cassava4.1_000058m.g	NB-ARC domain-containing disease resistance protein	0.2	>0.05	-0.2	>0.05	0.5	>0.05	1.1	2.92E-03	-0.2	>0.05	0.0	>0.05	-0.7	>0.05
**PR**															
Cassava4.1_012383m.g	Pathogenesis-related thaumatin superfamily protein	1.1	>0.05	0.2	>0.05	2.5	2.88E-02	3.5	2.48E-03	0.6	>0.05	0.4	>0.05	1.5	1.70E-02
Cassava4.1_011960m.g	Pathogenesis-related thaumatin superfamily protein	1.1	>0.05	0.5	>0.05	-2.2	>0.05	1.4	>0.05	2.3	1.62E-03	-0.8	>0.05	0.7	>0.05
**LEA**															
Cassava4.1_025676m.g	Late embryogenesis abundant protein, group 2	0.4	>0.05	1.0	>0.05	1.2	2.89E-02	1.4	3.32E-06	-0.1	>0.05	-0.3	>0.05	-0.1	>0.05
Cassava4.1_019959m.g	Late Embryogenesis Abundant 4-5	-1.0	>0.05	0.0	>0.05	2.3	>0.05	2.3	1.28E-05	5.4	>0.05	0.3	>0.05	-0.4	>0.05
**WRKY**															
Cassava4.1_014614m.g	WRKY DNA-binding protein 40	3.0	>0.05	-0.6	>0.05	-2.2	>0.05	4.0	4.54E-03	1.7	>0.05	-3.5	>0.05	-3.3	1.70E-07
Cassava4.1_011680m.g	WRKY DNA-binding protein 70	1.3	>0.05	-0.1	>0.05	-2.2	2.08E-02	2.1	1.16E-02	2.2	>0.05	-1.9	>0.05	-1.8	>0.05
**Heat shock proteins**															
Cassava4.1_033681m.g	Heat shock protein 90.1	0.6	>0.05	1.9	>0.05	4.7	>0.05	2.0	3.67E-02	-0.6	>0.05	-0.2	>0.05	-0.6	>0.05
Cassava4.1_034243m.g	Heat-shock protein 70T-2	-0.3	>0.05	1.1	>0.05	3.9	>0.05	0.0	>0.05	0.0	>0.05	0.3	>0.05	0.3	>0.05
Cassava4.1_003240m.g	Heat shock protein 70B	1.5	>0.05	2.2	>0.05	3.6	>0.05	-1.3	>0.05	1.1	>0.05	1.5	>0.05	0.9	>0.05

Cassava4.1_014648m.g	Heat shock protein 21	2.0	>0.05	2.0	>0.05	7.6	1.58E-01	1.6	>0.05	1.0	>0.05	-0.6	>0.05	-3.8	>0.05
Cassava4.1_009750m.g	Heat shock transcription factor A2	-0.4	>0.05	1.3	>0.05	6.1	1.35E-03	7.3	2.78E-03	-0.5	>0.05	-1.4	>0.05	0.0	>0.05
Cassava4.1_010803m.g	Heat shock transcription factor A6B	0.7	>0.05	2.0	>0.05	5.4	6.80E-05	3.4	7.36E-04	-2.8	>0.05	-0.1	>0.05	0.0	>0.05
Cassava4.1_003144m.g	Heat shock protein 70 (HSP 70) family protein	1.0	>0.05	-0.2	>0.05	0.8	4.41E-02	2.1	>0.05	-0.6	>0.05	-0.7	>0.05	-0.3	>0.05
Cassava4.1_001300m.g	Heat shock protein 101	0.4	>0.05	2.0	>0.05	1.5	>0.05	-0.2	>0.05	0.8	>0.05	0.5	>0.05	1.4	>0.05
Cassava4.1_007779m.g	DNAJ heat shock N-terminal domain-containing protein	0.7	>0.05	1.6	>0.05	0.8	>0.05	1.2	3.97E-04	-0.8	>0.05	0.2	>0.05	0.1	>0.05
Cassava4.1_008997m.g	HSP70-interacting protein 1	0.4	>0.05	0.9	>0.05	1.8	1.53E-07	0.8	1.61E-02	0.0	>0.05	-0.4	>0.05	0.5	>0.05
Cassava4.1_001924m.g	Heat shock protein 89.1	0.3	>0.05	2.1	>0.05	1.3	>0.05	2.4	2.06E-14	-0.1	>0.05	-0.7	>0.05	-0.2	>0.05
Cassava4.1_002706m.g	Chloroplast heat shock protein 70-2	0.0	>0.05	0.8	>0.05	0.1	>0.05	0.8	6.52E-03	-0.5	>0.05	-0.2	>0.05	-0.1	>0.05
Cassava4.1_001607m.g	Heat shock protein 70 (HSP 70) family protein	0.2	>0.05	2.1	>0.05	0.9	>0.05	1.2	7.00E-06	-0.5	>0.05	-0.2	>0.05	0.2	>0.05
Cassava4.1_003340m.g	Heat shock protein 70	0.9	>0.05	1.7	>0.05	0.9	>0.05	1.0	2.46E-02	0.1	>0.05	-0.1	>0.05	-0.7	>0.05
**Chaperones**															
Cassava4.1_018127m.g	HSP20-like chaperones superfamily protein	1.0	>0.05	1.8	>0.05	4.1	>0.05	1.3	>0.05	-0.4	>0.05	-0.5	>0.05	-0.9	>0.05
Cassava4.1_020888m.g	Trigger factor type chaperone family protein	-0.2	>0.05	2.0	6.39E-04	2.4	1.60E-04	0.8	>0.05	0.1	>0.05	0.3	>0.05	0.0	>0.05
Cassava4.1_008620m.g	Trigger factor type chaperone family protein	-0.2	>0.05	1.7	3.55E-03	2.0	1.79E-07	0.8	1.53E-02	0.1	>0.05	0.1	>0.05	-0.2	>0.05
Cassava4.1_001921m.g	Chaperone protein htpG family protein	0.1	>0.05	2.0	>0.05	1.4	1.82E-04	1.8	5.72E-12	-0.6	>0.05	-0.2	>0.05	-0.5	>0.05

Cassava4.1_018031m.g	Chaperone DNAJ-domain superfamily protein	0.2	>0.05	2.1	>0.05	3.2	2.18E-19	2.9	9.29E-28	0.4	>0.05	0.6	>0.05	0.4	>0.05
Cassava4.1_018200m.g	HSP20-like chaperones superfamily protein	0.0	>0.05	2.0	>0.05	5.7	>0.05	1.5	>0.05	0.9	>0.05	-0.4	>0.05	-1.7	>0.05
Cassava4.1_026342m.g	HSP20-like chaperones superfamily protein	2.7	>0.05	1.6	>0.05	8.6	>0.05	-1.1	>0.05	0.5	>0.05	-10.0	>0.05	-1.2	>0.05
Cassava4.1_001827m.g	Chaperone protein htpG family protein	1.4	>0.05	0.2	>0.05	2.0	1.81E-07	2.2	2.78E-18	-0.9	>0.05	-1.0	>0.05	-0.2	>0.05
Cassava4.1_014653m.g	Chaperone DNAJ-domain superfamily protein	0.8	>0.05	1.6	>0.05	3.9	4.07E-25	2.3	3.15E-15	-0.4	>0.05	0.3	>0.05	-1.7	>0.05
Cassava4.1_001905m.g	Chaperone protein htpG family protein	0.4	>0.05	2.0	>0.05	1.9	>0.05	1.0	1.41E-03	0.4	>0.05	0.0	>0.05	-0.6	>0.05
Cassava4.1_004263m.g	Chaperonin-60alpha	0.2	>0.05	1.7	>0.05	1.7	3.27E-02	1.2	2.03E-05	-0.6	>0.05	-0.4	>0.05	-0.2	>0.05
Cassava4.1_014410m.g	Chaperonin 20	0.0	>0.05	1.3	>0.05	1.3	1.82E-03	1.2	5.13E-05	-0.1	>0.05	-0.3	>0.05	-0.1	>0.05
Cassava4.1_018353m.g	Chaperone DNAJ-domain superfamily protein	1.7	>0.05	1.3	>0.05	2.9	6.91E-03	1.8	1.71E-04	-2.1	1.90E-03	0.4	>0.05	1.8	>0.05
**Transcription factors (NmrA, GATA and GRAS)**															
Cassava4.1_028589m.g	NmrA-like negative transcriptional regulator family protein	-0.4	>0.05	-0.2	>0.05	3.2	2.64E-04	3.5	8.63E-06	0.1	>0.05	0.5	>0.05	4.7	>0.05
Cassava4.1_032524m.g	NmrA-like negative transcriptional regulator family protein	-0.7	>0.05	1.2	>0.05	2.7	3.00E-03	2.2	3.63E-02	0.4	>0.05	0.6	>0.05	2.8	>0.05
Cassava4.1_032811m.g	GRAS family transcription factor	-0.1	>0.05	0.2	>0.05	3.2	4.52E-05	0.8	6.83E-03	0.0	>0.05	0.5	>0.05	-0.3	>0.05
Cassava4.1_016750m.g	GATA type zinc finger Transcription factor family protein	-0.2	>0.05	1.3	>0.05	2.0	>0.05	0.7	>0.05	-0.7	>0.05	-0.2	>0.05	-0.1	>0.05
Cassava4.1_011886m.g	GATA transcription factor 5	0.3	>0.05	0.3	>0.05	2.8	>0.05	1.7	1.70E-05	-0.8	>0.05	0.4	>0.05	0.4	>0.05
**NAC transcription factors**															

Cassava4.1_011029m.g	NAC domain containing protein 96	-1.5	>0.05	0.2	>0.05	1.0	>0.05	5.1	2.07E-06	0.1	>0.05	-2.0	>0.05	0.6	>0.05
Cassava4.1_015961m.g	NAC transcription factor-like 9	0.8	>0.05	1.3	>0.05	2.9	>0.05	1.0	>0.05	-0.6	>0.05	-5.8	>0.05	0.8	>0.05
Cassava4.1_023870m.g	NAC transcription factor-like 9	0.4	>0.05	1.0	>0.05	2.2	7.62E-04	1.2	>0.05	-1.1	>0.05	-2.7	>0.05	1.3	>0.05
**eIF**															
Cassava4.1_016601m.g	Eukaryotic initiation factor 4E protein	0.5	>0.05	-0.3	>0.05	0.4	>0.05	0.4	>0.05	-0.8	>0.05	-0.2	>0.05	0.3	>0.05

Log2foldchange (Log2FC) and level of significance (Adj p-values) of selected 55 genes. Most significant genes, and respective time points of significance are colored red. Genes colored blue were not significant after FDR correction of p-values but were selected based on their substantial fold change. VirtualPlant annotation in based on Arabidopsis genes as based on homology mapping using information provided in Phytozome.

Supplementary Table S7: Expression ratios in cassava variety Albert of 55 manually selected genes that belong to defense-related gene families**.** These are the same 55 genes as shown in Supplementary Table S6, but none of them was statistically significantly differentially expressed at any time point in Albert.

Gene ID	Gene annotation based VirtualPlant*	6 hag		1 dag		2 dag		5 dag		8 dag		45 dag		54 dag	
		Log2 FC	Adj Pval	Log2 FC	Adj Pval	Log2 FC	Adj Pval	Log2 FC	Adj Pval	Log2 FC	Adj Pval	Log2 FC	Adj Pval	Log2 FC	Adj Pval
**LRR**															
Cassava4.1_001048m.g	Leucine-rich repeat transmembrane protein kinase	0.3	>0.05	-0.4	>0.05	0.1	>0.05	0.1	>0.05	-0.3	>0.05	2.6	>0.05	0.6	>0.05
Cassava4.1_034154m.g	Leucine-rich repeat (LRR) family protein	-0.9	>0.05	0.6	>0.05	0.6	>0.05	0.2	>0.05	-0.1	>0.05	-0.5	>0.05	-0.8	>0.05
Cassava4.1_007501m.g	Leucine-rich repeat (LRR) family protein	0.2	>0.05	-1.1	>0.05	0.2	>0.05	0.1	>0.05	0.2	>0.05	2.2	>0.05	-0.1	>0.05
Cassava4.1_000978m.g	Leucine-rich receptor-like protein kinase family protein	-0.8	>0.05	0.0	>0.05	-0.1	>0.05	-0.7	>0.05	0.3	>0.05	0.5	>0.05	-0.2	>0.05
Cassava4.1_000765m.g	Leucine-rich repeat transmembrane protein kinase	-0.7	>0.05	-0.2	>0.05	-0.8	>0.05	0.2	>0.05	0.0	>0.05	-0.5	>0.05	-1.2	>0.05
**NBARC**															
Cassava4.1_029764m.g	NB-ARC domain-containing disease resistance protein	-0.1	>0.05	-0.6	>0.05	0.2	>0.05	-0.5	>0.05	0.0	>0.05	0.3	>0.05	0.0	>0.05
Cassava4.1_022732m.g	NB-ARC domain-containing disease resistance protein	-0.4	>0.05	-0.3	>0.05	0.1	>0.05	0.1	>0.05	0.0	>0.05	0.0	>0.05	0.1	>0.05
Cassava4.1_022172m.g	NB-ARC domain-containing disease resistance protein	-0.2	>0.05	-0.2	>0.05	0.3	>0.05	0.7	>0.05	0.1	>0.05	-0.3	>0.05	-0.2	>0.05

Cassava4.1_001752m.g	NB-ARC domain-containing disease resistance protein	-0.3	>0.05	-0.6	>0.05	0.2	>0.05	-1.0	>0.05	0.0	>0.05	0.5	>0.05	-0.1	>0.05
Cassava4.1_034172m.g	NB-ARC domain-containing disease resistance protein	-0.3	>0.05	-0.5	>0.05	0.5	>0.05	0.6	>0.05	0.2	>0.05	0.2	>0.05	0.0	>0.05
Cassava4.1_000058m.g	NB-ARC domain-containing disease resistance protein	-0.3	>0.05	-0.4	>0.05	0.3	>0.05	-0.1	>0.05	0.0	>0.05	0.8	>0.05	-0.1	>0.05
**PR**															
Cassava4.1_012383m.g	Pathogenesis-related thaumatin superfamily protein	-0.3	>0.05	1.4	>0.05	0.4	>0.05	-3.0	>0.05	0.6	>0.05	1.7	>0.05	-0.5	>0.05
Cassava4.1_011960m.g	Pathogenesis-related thaumatin superfamily protein	-1.9	>0.05	-1.0	>0.05	-0.5	>0.05	0.3	>0.05	0.1	>0.05	-0.3	>0.05	-0.7	>0.05
**LEA**															
Cassava4.1_025676m.g	Late embryogenesis abundant protein, group 2	-0.1	>0.05	0.2	>0.05	-0.4	>0.05	-0.3	>0.05	-0.1	>0.05	-0.2	>0.05	-0.4	>0.05
Cassava4.1_019959m.g	Late Embryogenesis Abundant 4-5	-0.1	>0.05	0.4	>0.05	0.5	>0.05	-0.1	>0.05	-0.6	>0.05	-0.5	>0.05	1.1	>0.05
**WRKY**															
Cassava4.1_014614m.g	WRKY DNA-binding protein 40	-3.5	>0.05	-2.4	>0.05	-1.6	>0.05	-1.1	>0.05	0.0	>0.05	-0.2	>0.05	-1.6	>0.05
Cassava4.1_011680m.g	WRKY DNA-binding protein 70	-0.9	>0.05	-1.2	>0.05	-0.3	>0.05	-2.1	>0.05	0.5	>0.05	0.0	>0.05	-0.6	>0.05
**Heat shock proteins**															
Cassava4.1_033681m.g	Heat shock protein 90.1	-0.9	>0.05	1.1	>0.05	1.0	>0.05	0.9	>0.05	-0.5	>0.05	-1.3	>0.05	-0.1	>0.05
Cassava4.1_034243m.g	Heat-shock protein 70T-2	-0.3	>0.05	0.8	>0.05	1.1	>0.05	-0.4	>0.05	-0.8	>0.05	-1.2	>0.05	0.4	>0.05
Cassava4.1_003240m.g	Heat shock protein 70B	-0.1	>0.05	0.9	>0.05	1.6	>0.05	0.6	>0.05	-1.2	>0.05	0.0	>0.05	0.7	>0.05
Cassava4.1_014648m.g	Heat shock protein 21	0.1	>0.05	0.5	>0.05	1.4	>0.05	3.5	>0.05	-2.7	>0.05	-0.9	>0.05	2.8	>0.05

Cassava4.1_009750m.g	Heat shock transcription factor A2	-1.1	>0.05	0.3	>0.05	-0.6	>0.05	-0.3	>0.05	0.0	>0.05	-1.2	>0.05	0.9	>0.05
Cassava4.1_010803m.g	Heat shock transcription factor A6B	1.9	>0.05	1.8	>0.05	2.2	>0.05	-0.1	>0.05	0.7	>0.05	-0.3	>0.05	0.9	>0.05
Cassava4.1_003144m.g	Heat shock protein 70 (Hsp 70) family protein	-0.8	>0.05	0.1	>0.05	-0.2	>0.05	-1.0	>0.05	0.0	>0.05	-0.1	>0.05	-0.3	>0.05
Cassava4.1_001300m.g	Heat shock protein 101	-0.5	>0.05	1.0	>0.05	0.2	>0.05	0.3	>0.05	0.1	>0.05	-0.8	>0.05	-0.6	>0.05
Cassava4.1_007779m.g	DNAJ heat shock N-terminal domain-containing protein	-0.7	>0.05	0.8	>0.05	0.3	>0.05	0.0	>0.05	0.2	>0.05	-0.5	>0.05	-0.2	>0.05
Cassava4.1_008997m.g	HSP70-interacting protein 1	-0.2	>0.05	0.3	>0.05	-0.3	>0.05	0.1	>0.05	-0.1	>0.05	-0.3	>0.05	0.1	>0.05
Cassava4.1_001924m.g	Heat shock protein 89.1	-0.3	>0.05	0.4	>0.05	-0.2	>0.05	0.3	>0.05	-0.3	>0.05	-0.9	>0.05	0.0	>0.05
Cassava4.1_002706m.g	Chloroplast heat shock protein 70-2	-0.1	>0.05	0.4	>0.05	0.1	>0.05	-0.4	>0.05	0.1	>0.05	0.0	>0.05	-0.2	>0.05
Cassava4.1_001607m.g	Heat shock protein 70 (Hsp 70) family protein	-0.6	>0.05	0.8	>0.05	0.1	>0.05	0.1	>0.05	0.1	>0.05	-0.2	>0.05	-0.4	>0.05
Cassava4.1_003340m.g	Heat shock protein 70	-0.9	>0.05	0.8	>0.05	0.6	>0.05	0.0	>0.05	-0.2	>0.05	-0.6	>0.05	-0.6	>0.05
**Chaperones**															
Cassava4.1_018127m.g	HSP20-like chaperones superfamily protein	0.3	>0.05	0.4	>0.05	0.4	>0.05	0.0	>0.05	-0.4	>0.05	-0.4	>0.05	0.6	>0.05
Cassava4.1_020888m.g	Trigger factor type chaperone family protein	0.1	>0.05	0.3	>0.05	0.3	>0.05	0.1	>0.05	-0.1	>0.05	0.2	>0.05	0.0	>0.05
Cassava4.1_008620m.g	Trigger factor type chaperone family protein	0.3	>0.05	0.3	>0.05	0.2	>0.05	0.1	>0.05	0.2	>0.05	0.2	>0.05	0.0	>0.05
Cassava4.1_001921m.g	Chaperone protein htpG family protein	0.2	>0.05	0.4	>0.05	-0.3	>0.05	-0.1	>0.05	-0.1	>0.05	-0.2	>0.05	0.1	>0.05
Cassava4.1_018031m.g	Chaperone DNAJ-domain superfamily protein	-0.2	>0.05	0.4	>0.05	0.5	>0.05	-0.7	>0.05	0.0	>0.05	0.8	>0.05	-0.1	>0.05

Cassava4.1_018200m.g	HSP20-like chaperones superfamily protein	0.4	>0.05	1.0	>0.05	2.1	>0.05	1.5	>0.05	-0.9	>0.05	-1.1	>0.05	0.5	>0.05
Cassava4.1_026342m.g	HSP20-like chaperones superfamily protein	2.2	>0.05	0.8	>0.05	4.5	>0.05	-0.6	>0.05	-1.5	>0.05	-10.0	>0.05	1.9	>0.05
Cassava4.1_001827m.g	Chaperone protein htpG family protein	-1.0	>0.05	0.0	>0.05	-0.6	>0.05	-0.5	>0.05	-0.1	>0.05	-0.2	>0.05	-0.3	>0.05
Cassava4.1_014653m.g	Chaperone DNAJ-domain superfamily protein	0.7	>0.05	0.8	>0.05	0.6	>0.05	0.4	>0.05	0.3	>0.05	0.8	>0.05	0.1	>0.05
Cassava4.1_001905m.g	Chaperone protein htpG family protein	0.4	>0.05	0.7	>0.05	-0.1	>0.05	0.6	>0.05	-0.2	>0.05	0.0	>0.05	0.2	>0.05
Cassava4.1_004263m.g	Chaperonin-60alpha	0.1	>0.05	0.5	>0.05	-0.1	>0.05	-0.2	>0.05	-0.1	>0.05	0.1	>0.05	-0.1	>0.05
Cassava4.1_014410m.g	Chaperonin 20	0.0	>0.05	0.3	>0.05	-0.3	>0.05	-0.5	>0.05	-0.1	>0.05	-0.1	>0.05	0.0	>0.05
Cassava4.1_018353m.g	Chaperone DNAJ-domain superfamily protein	-2.1	>0.05	0.6	>0.05	0.6	>0.05	0.0	>0.05	0.2	>0.05	-0.2	>0.05	-0.1	>0.05
**Transcription factors (NmrA, GATA and GRAS)**															
Cassava4.1_028589m.g	NmrA-like negative transcriptional regulator family protein	-2.1	>0.05	-1.4	>0.05	0.5	>0.05	-1.5	>0.05	0.2	>0.05	4.5	>0.05	-1.1	>0.05
Cassava4.1_032524m.g	NmrA-like negative transcriptional regulator family protein	-4.0	>0.05	-1.6	>0.05	-0.3	>0.05	-3.6	>0.05	0.7	>0.05	3.5	>0.05	0.1	>0.05
Cassava4.1_032811m.g	GRAS family transcription factor	0.4	>0.05	0.1	>0.05	0.6	>0.05	-0.1	>0.05	0.2	>0.05	-0.2	>0.05	0.6	>0.05
Cassava4.1_016750m.g	GATA type zinc finger transcription factor family protein	0.2	>0.05	0.2	>0.05	0.4	>0.05	0.3	>0.05	0.1	>0.05	0.2	>0.05	-0.1	>0.05
Cassava4.1_011886m.g	GATA transcription factor 5	0.0	>0.05	-0.5	>0.05	0.6	>0.05	-0.9	>0.05	0.2	>0.05	-0.1	>0.05	0.2	>0.05
**NAC transcription factors**															
Cassava4.1_011029m.g	NAC domain containing protein 96	-0.7	>0.05	-0.1	>0.05	0.4	>0.05	-2.4	>0.05	-0.5	>0.05	-0.4	>0.05	-0.7	>0.05

Cassava4.1_015961m.g	NAC transcription factor-like 9	0.6	>0.05	-1.4	>0.05	0.4	>0.05	0.4	>0.05	-3.0	>0.05	-2.0	>0.05	-3.2	>0.05
Cassava4.1_023870m.g	NAC transcription factor-like 9	0.3	>0.05	0.2	>0.05	0.6	>0.05	-1.9	>0.05	-1.0	>0.05	-0.5	>0.05	-0.4	>0.05
**eIF**															
Cassava4.1_016601m.g	Eukaryotic initiation factor 4E protein	-0.5	>0.05	0.0	>0.05	0.1	>0.05	-0.5	>0.05	-0.4	>0.05	-0.7	>0.05	-0.1	>0.05
		0.0	>0.05	0.5	>0.05	0.1	>0.05	0.0	>0.05	0.2	>0.05	0.1	>0.05	-0.1	>0.05

Log2foldchange (Log2FC) and level of significance (Adj p-values) (Adj Pval) of selected 55 genes.
